# LINC00958 promotes the proliferation of TSCC via miR-211-5p/CENPK axis and activating the JAK/STAT3 signaling pathway

**DOI:** 10.1186/s12935-021-01808-z

**Published:** 2021-03-03

**Authors:** Bo Jia, Junfeng Dao, Jiusong Han, Zhijie Huang, Xiang Sun, Xianghuai Zheng, Shijian Xiang, Huixi Zhou, Shuguang Liu

**Affiliations:** 1 Department of Stomatology, Shunde Hospital, Southern Medical University, Foshan, Guangdong 528300 China; 2 Department of Oral Surgery, Stomatological Hospital, Southern Medical University, Guangzhou, 510280 China; 3 Department of Prosthodontics, Stomatological Hospital, Southern Medical University, Guangzhou, 510280 China; 4 Department of Pharmacy, Seventh Affiliated Hospital of Sun Yat-Sen University, Shenzhen, 518107 China

**Keywords:** TSCC, LINC00958, miR-211-5p, CENPK, Proliferation

## Abstract

**Background:**

Tongue squamous cell carcinoma (TSCC) is one of the most common oral tumors. Recently, long intergenic noncoding RNA 00958 (LINC00958) has been identified as an oncogene in human cancers. Nevertheless, the role of LINC00958 and its downstream mechanisms in TSCC is still unknown.

**Methods:**

The effect of LINC00958 on TSCC cells proliferation and growth were assessed by CCK-8, colony formation, 5-Ethynyl-2′-deoxyuridline (EdU) assay and flow cytometry assays in vitro and tumor xenograft model in vivo. Bioinformatics analysis was used to predict the target of LINC00958 in TSCC, which was verified by RNA immunoprecipitation and luciferase reporter assays.

**Results:**

LINC00958 was increased in TSCC tissues, and patients with high LINC00958 expression had a shorter overall survival. LINC00958 knockdown significantly decreased the growth rate of TSCC cells both in vitro and in vivo. In mechanism, LINC00958 acted as a ceRNA by competitively sponging miR-211-5p. In addition, we identified CENPK as a direct target gene of miR-211-5p, which was higher in TSCC tissues than that in adjacent normal tissues. Up-regulated miR-211-5p or down-regulated CENPK could abolish LINC00958-induced proliferation promotion in TSCC cells. Furthermore, The overexpression of CENPK promoted the expression of oncogenic cell cycle regulators and activated the JAK/STAT3 signaling.

**Conclusions:**

Our findings suggested that LINC00958 is a potential prognostic biomarker in TSCC.

## Introduction

Tongue squamous cell carcinoma is one of the most frequently diagnosed malignancies in the oral cavity [1, 2]. Because of its high metastatic and proliferative ability, TSCC is a considerable threat to human health worldwide [3, 4]. It caused about 12,060 new cases and 2030 deaths in the United States in 2011 [5], and about 48,100 new cases and 22,100 deaths were recorded in China in 2015 [1]. Over the past decades, new developments have been achieved in the therapeutic management of TSCC, such as surgery, chemotherapy and radiotherapy; however, the five-year survival rate of TSCC patients is still less than 50% [3, 6]. Therefore, increased understanding of the complex mechanisms of TSCC cells is imperative to the development of effective and mechanism-based therapeutic modalities for this malignancy.

Increasing evidence has proved that Long non-coding RNAs (lncRNAs) modulate diverse physiologic and pathologic processes, including proliferation, migration, invasion, and autophagy[7]. It has been reported as a core regulator in the processes of chromatin modification, transcriptional regulation, alternative splicing and interaction with RNAs and protein complex at transcriptional or posttranscriptional levels[8]. LncRNA-SRLR is responsible for the sorafenib tolerance in renal cell carcinoma via binding to NF-κB and promotes IL-6 transcription[9]. LncRNA NORAD promotes hepatocellular carcinoma progression, it functions as a ceRNA to target miR‐202‐5p,leading to the enhanced activation of TGF‐β pathway[10]. H19 facilitated TSCC migration and invasion by sponging miR-let-7 [11] and lncRNA KCNQ1OT1 regulated proliferation and cisplatin resistance in tongue cancer via miR-211-5p-mediated Ezrin/Fak/Src signaling [12]. It is reported that High expression of LINC00958 is associated with malignancy, proliferation and poor prognosis in various cancers, such as oral squamous cell carcinoma, head and neck squamous cell carcinoma, and pancreatic cancer [13, 14] suggesting that LINC00958 is closely connected with cancer development. However, its role and the associated downstream mechanisms in TSCC have remained unknown.

MicroRNAs (miRNAs) are evolutionarily conserved small RNAs (20–22 nucleotides long) without protein coding potential. MiRNAs can negatively regulate gene expression post-transcriptionally by binding to complementary sequences on their target mRNAs [15, 16]. A previous study has reported that miR-3064 could promote pancreatic cancer cell growth, invasion, and sphere formation by down-regulating the levels of PIP4K2B—a tumor suppressor [17]. Some studies have indicated that miR-211-5p-mediated inhibition of SNAIL1 expression contributes to the suppression of gastric carcinoma progression [18]. MiR-211-5p has been linked to short survival in hepatocellular carcinoma patients, and the restoration of miR-211-5p expression inhibited hepatocellular carcinoma cell proliferation, migration and invasion in vitro by targeting ZEB2[19]. However, little is known about the role of miR-211-5p in TSCC.

In this study, lncRNAs in TSCC tissues from different patients at different stages were screened based on TCGA database analysis, and we identified that LINC00958 was the most up-regulated lncRNA in the TSCC tissues. In addition, we found that high LINC00958 expression was closely related to poor prognosis of TSCC patients. In vitro and in vivo functional experiments showed that LINC00958 promoted TSCC cell proliferation. Furthermore, LINC00958 sponged miR-211-5p to increase CENPK expression and activate the JAK/STAT3 pathway, and thus mediated proliferation. Our study elucidated the clinical significance and regulatory mechanism of LINC00958 in TSCC and provides a prognostic indicator as well as a promising therapeutic target for TSCC patients.

## Materials and methods

### RNA sequence data analysis

RNASeq and miRNASeq data from TSCC samples were downloaded from the Cancer Genome Atlas (TCGA) database (https://cancergenome.nih.gov/). All the data are publicly available. lncRNAs were identified according to the Ensembl database (http://www.ensembl.org/index.html, version 89) lncRNAs that were not included in this database were excluded. The edgeR package (Robinson, McCarthy & Smyth, 2010) was used to normalize gene expression of mRNAs (DEmRNAs), miRNAs (DEmiRNAs), and lncRNAs (DElncRNAs) in TSCC and normal tissues. Absolute log2FC ≥ 2 and FDR < 0.01 were used as cut-off criteria. Survival R package were further used to exclude the differentially expressed lncRNA without OS at P-value < 0.05. Next, the screened lncRNAs were used to predict lncRNA-miRNA interactions according to TargetScan. lncRNAs included in these interactions were used for further study.

### Clinical specimens

Patients with TSCC, who were diagnosed, treated, and followed up at the Department of Oral Surgery, Stomatological Hospital, Southern Medical University, were included in the study. This study was approved by the hospital institutional review board and written informed consent was obtained from all the patients. All the protocols were reviewed by the Joint Ethics Committee of the Southern Medical University and performed following national guidelines. Tissue samples were collected at surgery, immediately frozen in liquid nitrogen, and stored until total RNA or proteins were extracted.

### Cell lines and culture

HOK and five TSCC cell lines (CAL-27, SCC-9, SCC-4, SCC-15, SCC-25) were obtained from the Tumor Cell Bank of the Chinese Academy of Medical Science (Shanghai, China). CAL-27 cells were maintained in DMEM medium (Gibco, Grand Island, USA), which was supplemented with 10% fetal bovine serum (FBS, Gibco, Grand Island, USA), and the other cells were cultured in RPMI-1640 (Gibco, Grand Island, USA) supplemented with 10% FBS. For all cell lines, 100 IU/ml penicillin and 100 μg/mL streptomycin were added to the culture medium, and all of the cells were incubated at 37℃ in a humidified atmosphere of 95% air 5% CO_2_.

### Real-time quantitative PCR (RT-qPCR)

Total RNA was isolated from cells or tissues using TRIzol reagent (Invitrogen, California, USA) and then was converted to cDNA using a PrimeScript RT reagent kit (TaKaRa, Tokyo, Japan). RT-qPCR analysis were carried out in triplicate for each sample using SYBR Green Master Mix (TaKaRa, Tokyo, Japan). All primers are listed in Additional file 1: Table S1, and glyceraldehyde-3-phosphate dehydrogenase (GAPDH) served as the endogenous control. For detecting miRNA expression level, cDNA was synthesized using MicroRNAs qPCR Kit (SYBR Green Method) (Sangon Biotech, Shanghai, China), and U6 small nuclear RNA served as the endogenous control.

### CCK-8 assay

Cell viability was determined using the CCK-8 assay. Briefly, 2 × 10^3^ cells/well were seeded into 96-well plates, and the absorptions of the cells were measured using a CCK-8 kit (Dojindo, Kyushu, Japan) according to the manufacturer’s instructions at different indicated time points. Data were from three separate experiments with four replications each time.

### Clone formation assay

From each group, nearly 1 × 10^3^ cells were plated in each well of a 6-well culture plate. Each cell group consisted of three wells. The cells were incubated at 37 ℃ for 14 days with growth media being replaced every third day. Then, the cells were washed twice with PBS and stained with 0.5% crystal violet.

### EdU incorporation assays

Cells were cultured in 24-well plates, and 10 μM EdU was added to each well. Then, the cells were cultured for 2 h at 37 ℃ and were fixed with 4% formaldehyde for 20 min at RT. After washing with PBS, the incorporated EdU was detected with a kFluor488-EdU kit (KeyGEN, Jiangsu, China) for 30 min at RT, and subsequently, the cells were stained with Hoechst 33342 for 20 min and were captured using a fluorescence microscope (Olympus, Tokyo, Japan) and were merged using Adobe Photoshop 6.0 software. All experimental procedures were repeated at least three times.

### Cell cycle analysis by flow cytometry

Cell cycle analysis were performed using the Cell Cycle Analysis Kit (Beyotime, Jiangsu, China) as per the manufacturer’s instructions. Cells were harvested and fixed in 70% ethanol overnight at 4 ℃. Then, the cells were stained with 25 μg/mL propidium iodide containing 1 μg/mL RNase at 37 ℃ for 30 min. The cells were analyzed for their distribution in different phases of the cell cycle on FACSCalibur flow cytometer using CellQuestPro software (Becton Dickinson, New Jersey USA).

### RNA immunoprecipitation (RIP)

A Thermo Scientific RIP kit (Thermo, Waltham, MA, USA) was used to carry out RIP according to the manufacturer’s instructions. CAL-27 and SCC-9 cells were transfected with miR-211-5p mimics or mimics NC. Complete RIP lysis buffer was used to lyse cells. Magnetic beads conjugated with anti-Argonaute 2 (AGO2, Millipore, Massachusetts, USA) or control anti-immunoglobulin G (IgG, Abcam, Cambridge, England) antibody were used to incubate the cell extract. The cell extract was incubated for 6 h at 4℃. As the protein beads were removed, RT-qPCR was conducted for the purification of RNA.

### Western blot analysis

Cell lysates was separated by SDS–polyacrylamide gel (4–10%) electrophoresis, and then transferred to polyvinylidene fluoride (PVDF) membranes (Millipore, Massachusetts, USA). The membranes were then blocked with 5% skimmed milk and incubated overnight at 4 °C with the following primary detection antibodies: anti-CENPK (1:1000, Abcam, Cambridge, England), anti-C-MYC (1:1000, Abcam, England), anti-Cyclin D (1:1000, CST, Boston, USA), anti-Cyclin E (1:1000, Abcam, Cambridge, England), anti-Rb (1:1000, CST, Boston, USA), anti-p-Rb (1:1000, CST, Boston, USA), anti-GAPDH (1:10,000, Abcam, Cambridge, England), anti-JAK1 (1:1000, Abcam, Cambridge, England), anti-STAT3 (1:1000, Abcam, Cambridge, England), or anti-p-STAT3 (1:1000, Abcam, Cambridge, England). The species-matched secondary antibodies were then incubated for 1 h at room temperature and the proteins were detected using BeyoECLPlus (Beyotime, Jiangsu, China).

### Luciferase reporter assay

The wild-type or mutant LINC00958 fragment or CENPK 3′-UTR containing the predicted binding sites of miR-211-5p. were subcloned into a psiCHECK2 Dual-luciferase vector (Promega, Madison, USA). The luciferase reporter plasmids were co-transfected into TSCC cells with miR-211-5p mimics or the negative control. The relative luciferase activity was measured with the Dual-Luciferase Reporter Assay System (Promega, Madison, USA) according to the manufacturer’s instructions.

### IHC staining

We used anti-Ki67 antibody (CST, Boston, USA), anti-PCNA antibody (Abcam, Cambridge, England), anti-CENPK antibody (Abcam, Cambridge, England) and anti-p-STAT3 (CST, Boston, USA) to detect the expression of relatived proteins in the mouse xenografts. The sections were visualized using a Nikon ECLIPSE Ti microscope system and were processed with Nikon software.

### Plasmids, virus production, siRNA, and transfection

The full length of LINC00958 and shRNAs targeting LINC0058 was synthesized and cloned into the lentiviral plasmid pSin-EF2-puromycin (Addgene, Cambridge, MA, USA). pSin-EF2-LHX2-puromycin or negative control pSin-EF2-puromycin vector was then co-transfected into 293 T cells with the VSVG and PSPAX2 packaging plasmid (Addgene, Cambridge, MA, USA) using Lipofectamine 3000 reagent (Invitrogen). The supernatants were harvested and used to infect CAL-27 and SCC-9 cells, and stable clones were selected using 0.5 μg/mL puromycin. Small interfering RNA targeting CENPK (si-CENPK) was synthesized by RiboBio (Guangzhou, China). For CENPK knockdown, CAL-27 and SCC-9 were transfected with si-CENPK (50 nM) with Lipofectamine 3000 reagent (Invitrogen) according to the manufacturer’s instruction and harvested for assays (48 h after transfection). miR-211-5p mimics, miR-211-5p inhibitors and miR-controls were purchased from RiboBio (Guangzhou, China). MiRNA mimics, miRNA inhibitors, and miR-controls were transfected into cells at a concentration of 50 nM using Lipofectamine 3000 (Invitrogen, California, USA).

### In vivo nude mouse models

A total of twenty-four 5-week-old male nude mice were purchased from Guangdong Medical Laboratory Animal Center, and kept under specific pathogen-free conditions. Mice were divided into four groups at random and inoculated with Cal-27 and SCC-9 cells subcutaneously on the right flank with 2 × 10^6^ cells (n = 6 per experimental group). The growth of the tumors was observed and the volumes of tumors were measured every 4 days. The animals were sacrificed after 4 weeks of growth, and the tumors were excised for pathological examination.

### Subcellular fractionation and fluorescent in situ hybridization (FISH) assay

Nuclear and cytoplasmic RNA was separated with the NE-PER™ Nuclear and Cytoplasmic Extraction Reagents (Invitrogen) accordingly, and then analyzed by quantitative RT-PCR. FISH was performed for subcellular localization of LINC00958. The cover glasses were placed into a 24-well plate and cells were inoculated at the density of 6 × 10^4^ cells/well. When cell confluence reached 80%, the cover glasses were removed, and the cells were washed by PBS and fixed with 1 mL 4% paraformaldehyde at room temperature. The experiment was conducted according to the instructions of RiboTMlncRNA FISH Probe Mix (Red) (RiboBio Co., Ltd., Guangdong, China). The cells were observed and photographed under a fluorescence microscope.

### Statistical analysis

Data were expressed as mean ± SD of three independent experiments with GraphPad Prism software (version 6.0). Statistical analysis was performed using Statistical Package for Social Sciences (SPSS) software (version 16.0). Depending on the type of data, the appropriate statistical methods were used, including the t-test, one-way ANOVA, chi-square test and linear correlation analysis. The Kaplan–Meier method with the log-rank test was used to compare the survival rate of TSCC patients based on LINC00958 expression. Survival data were evaluated using univariate and multivariate Cox proportional hazards models. p < 0.05 was considered statistically significant.

## Results

### LINC00958 is specifically up-regulated in tongue cancer tissues and cell lines

**Fig. 1:**
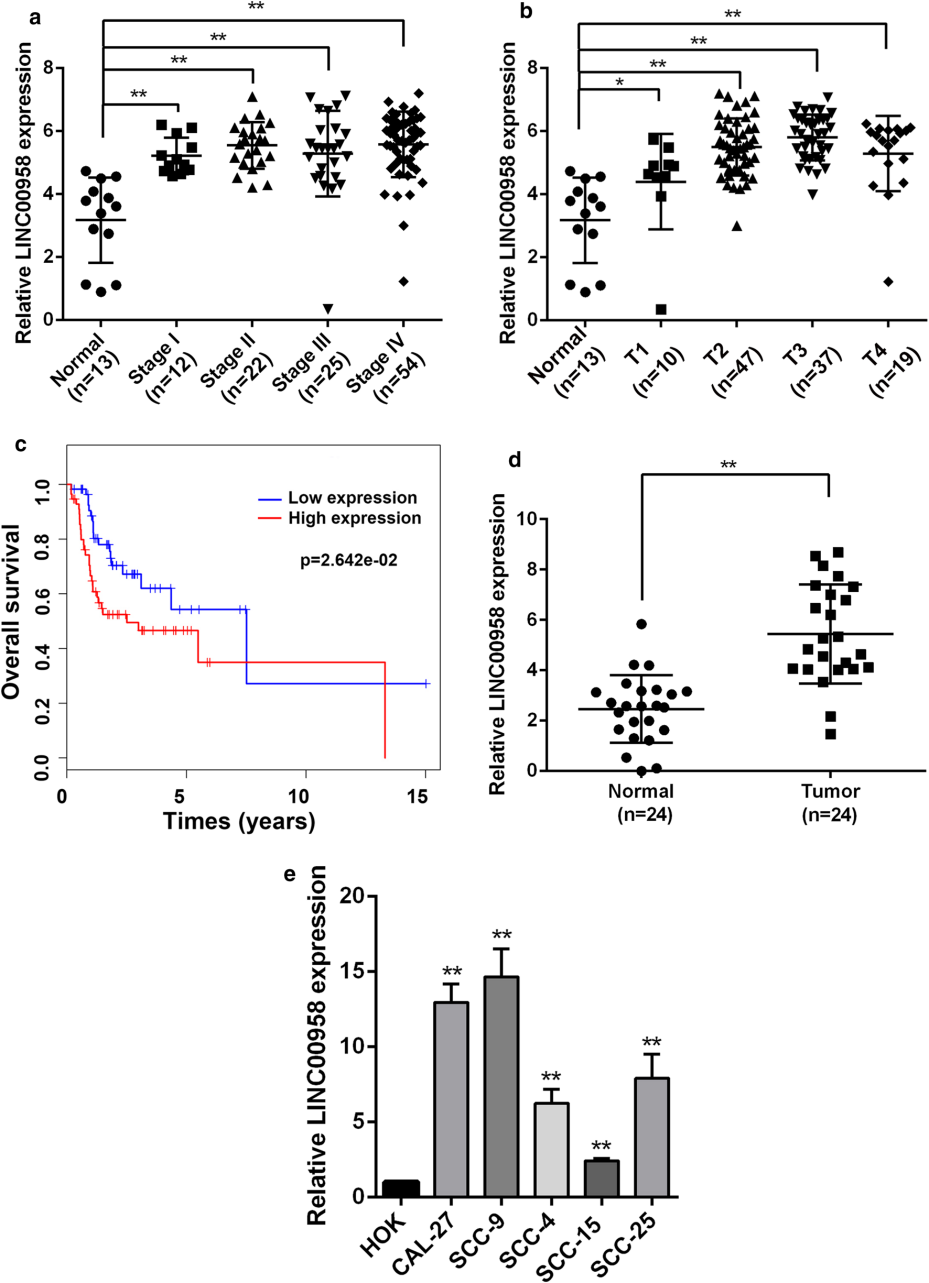
LINC00958 is specifically upregulated in tongue cancer tissues and tumor cell lines. **a** Relative expression of LINC00958 in normal tissues and different stages of TSCC tissues. Data were downloaded and analyzed from the TCGA database. **b** Relative expression of LINC00958 in normal tissues and different clinical T-staging of TSCC tissues. The data were downloaded and analyzed from the TCGA database. **c** Kaplan–Meier analysis of the correlation between LINC00958 expression and overall survival. The data were downloaded and analyzed from the TCGA database. **d** RT-qPCR analysis was applied to measure the expression of LINC00958 in TSCC tissues (n = 24) and normal tissues (n = 24) by RT-qPCR. **e** RT-qPCR analysis was applied to measure the expression of LINC00958 in HOK and 5 TSCC cell lines ^*^P < 0.05, ^**^P < 0.01, ^***^P < 0.001

Using TCGA database analysis, we detected aberrantly expressed lncRNAs between adjacent normal tissues and TSCC tissues from TSCC patients. Furthermore, we found that LINC00958 was highly up-regulated in tumor tissues, compared with normal tissues from the TCGA database, (Additional file 2: Figure S1a, b and Fig. 1a, b). Furthermore, the correlation between LINC00958 expression and clinicopathological traits was analyzed. LINC00958 expression was much higher in T3 and T4 than in T1 and T2 (Additional file 3: Table S2). In addition, Kaplan–Meier survival analysis indicated that increased LINC00958 expression was significantly associated with a lower rate of overall survival in TSCC patients (Fig. 1c), and high expression of LINC00958 predicted poor survival in some other solid tumors (Additional file 2: Figure S1C). We also examined the relative expression of LINC00958 in TSCC tissues (n = 24) and adjacent normal tissues (n = 24). RT-qPCR results showed that the expression of LINC00958 was significantly increased in TSCC samples than in adjacent normal tissues (Fig. 1d). This change in LINC00958 expression was further evaluated in TSCC cell lines including SCC-15, SCC-25, SCC-4, SCC-9 and CAL-27. As shown in Fig. 1e, the expression level of LINC00958 was up-regulated in TSCC cell lines, compared with HOK cells. These findings indicated that LINC00958 was significantly up-regulated in TSCC and predicte poor survival.

### LINC00958 promotes the proliferation of TSCC cells and affecting cell cycle distribution

**Fig. 2:**
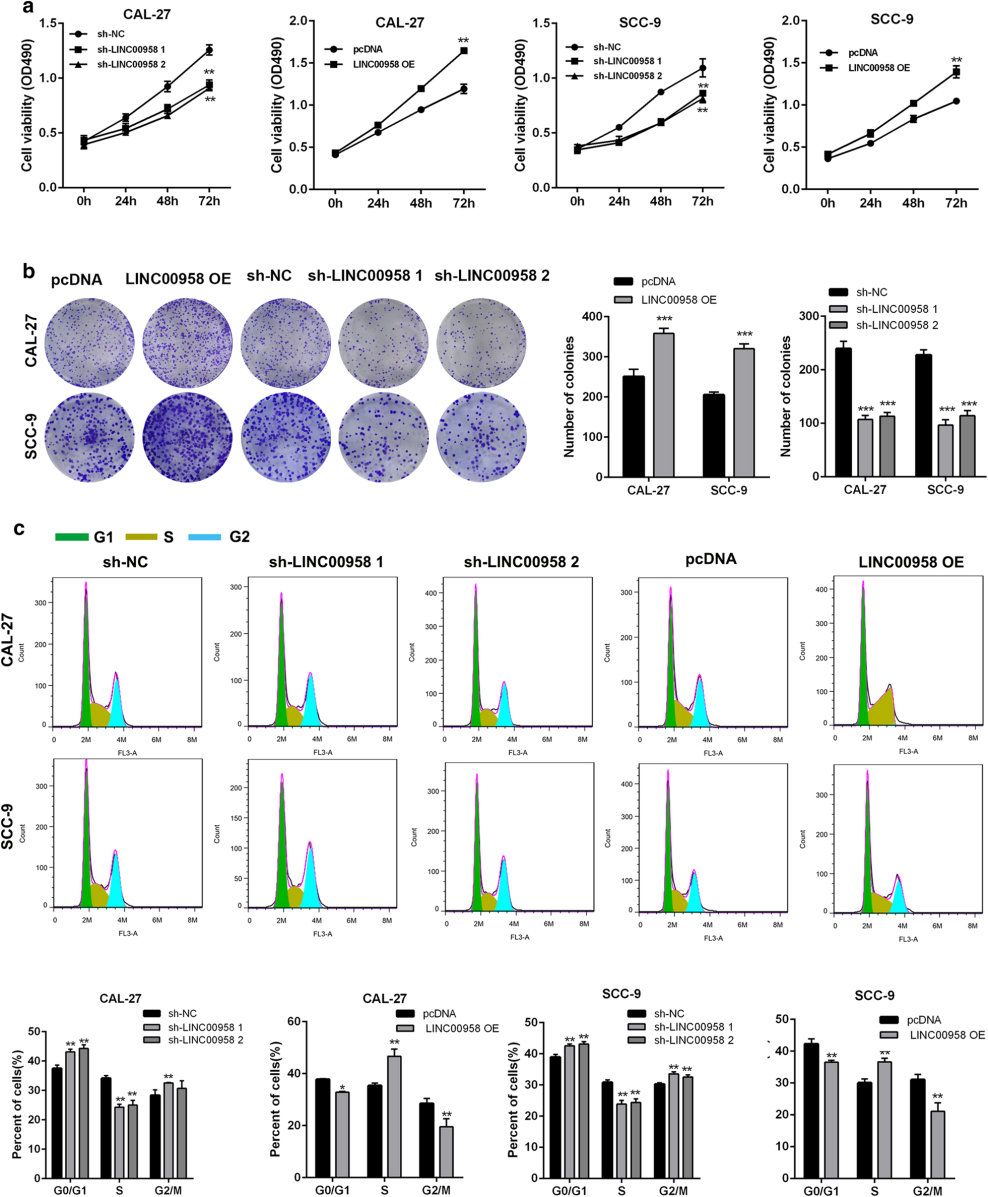
LINC00958 promotes the proliferation of TSCC cells and affecting cell cycle distribution. **a** CCK8 assay of CAL-27 and SCC-9 cells stably transfected with sh-LINC00958, pcDNA-LINC00958 and their corresponding control vectors. **b** Colony formation assay of CAL-27 and SCC-9 cells stably transfected with sh-LINC00958, pcDNA-LINC00958 and their corresponding control vectors. **c** Flow cytometric analysis was applied to measure the cell cycle distribution of CAL-27 and SCC-9 cells stably transfected with sh-LINC00958, pcDNA-LINC00958 and their corresponding control vectors. ^*^P < 0.05, ^**^P < 0.01, ^***^P < 0.001

To evaluate the influence of LINC00958 on TSCC cell growth, CAL-27 and SCC-9 cells were transfected with two different shRNAs against LINC00958 (sh-LINC00958-1 and sh-LINC00958-2), a LINC00958-overexpressing plasmid (pcDNA-LINC00958) and their corresponding controls. The transfection efficiency was confirmed by RT-qPCR analysis (Additional file 4: Figure S2A). CCK-8 assays and colon formation assays revealed a significant inhibition in the proliferation of TSCC cells transfected with sh-LINC00958, compared with those transfected with scrambled vectors, whereas the opposite effects were observed in TSCC cells with LINC00958 overexpression (Fig. 2a, b). In addition, the knockdown of LINC00958 decreased cell division, whereas overexpression of LINC00958 significantly increased cell division as shown by EdU assays (Additional file 4: Figure S2B). Morover, flow cytometry analysis indicated that overexpression of LINC00958 promoted cell cycle progression, while the knockdown of LINC00958 induced cell cycle arrest at the G0/G1 phase in CAL-27 and SCC-9 cells (Fig. 2c).Taken together, these findings suggested that LINC00958 enhances the cell proliferation ability of TSCC cells and promotes cell cycle progression.

### Down-regulation of LINC00958 suppresses TSCC tumor growth in vivo

**Fig. 3:**
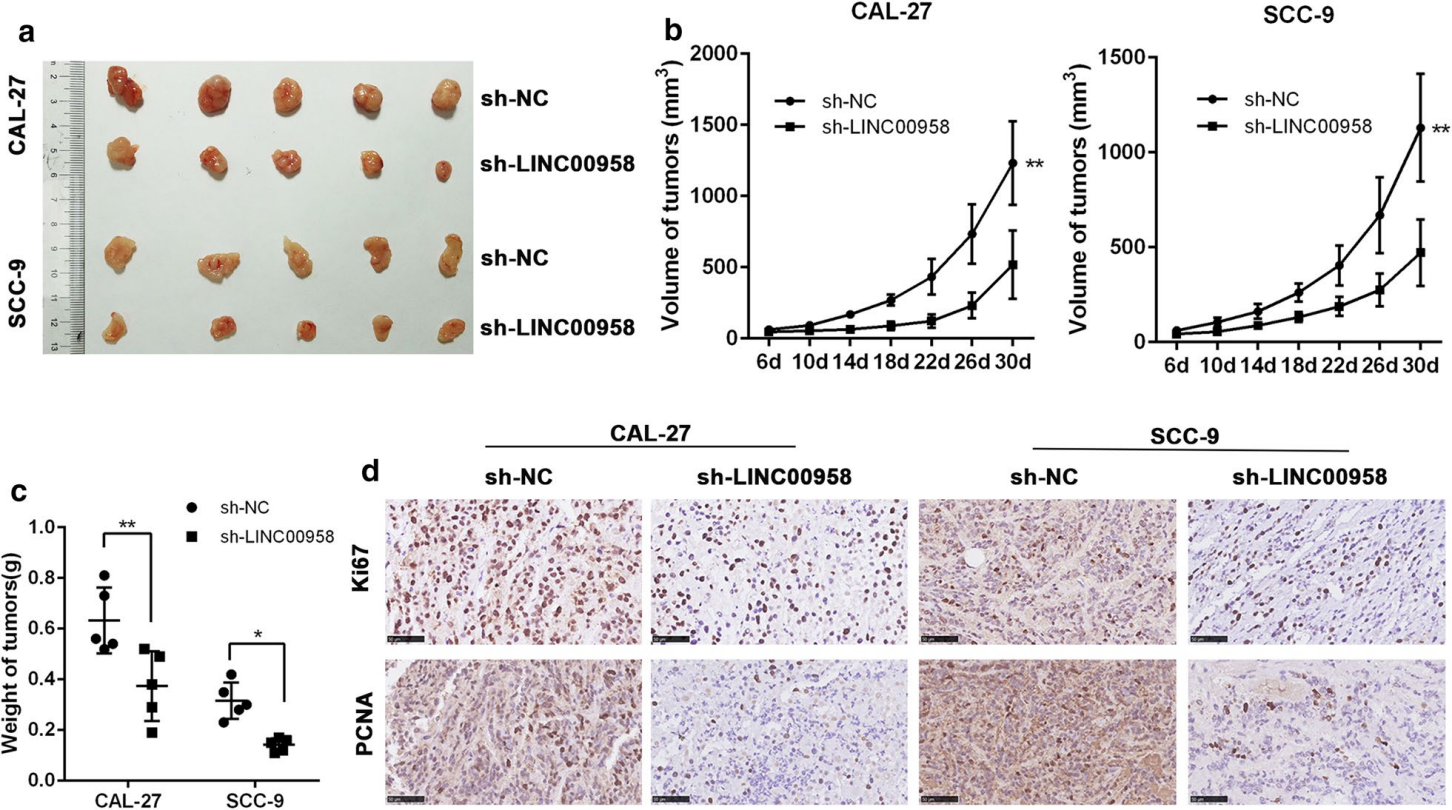
Downregulation of LINC00958 suppresses TSCC tumor growth in vivo. **a** Nude mice were transplanted subcutaneously with CAL-27 and SCC-9 cells stably transfected with sh-LINC00958 or the control shRNA. A representative picture of the morphology of tumor xenografts after excision at 30 days of treatment. **b** Tumor volumes were measured every 4 days. **c** Tumor weights were measured after the mice were sacrificed. **d** The expression of Ki67 and PCNA in the xenografts were examined by IHC. ^*^P < 0.05, ^**^P < 0.01, ^***^P < 0.001

To explore the effect of LINC0095 on the tumorigenesis of TSCC cells in vivo, CAL-27 cells with stably knockdown LINC00958 was subcutaneously transplanted into nude mice. The growth rate of tumors in the LINC00958 knockdown group was substantially suppressed compared with that in the control group (Fig. 3a, b). The tumor weights in the LINC00958 knockdown group were significantly lower than those of in the control group (Fig. 3c). Furthermore, LINC00958 knockdown reduced Ki-67 and proliferating cell nuclear antigen (PCNA) positivity as confirmed by immunohistochemistry results (Fig. 3d). These results indicated that LINC00958 knockdown inhibits TSCC cell growth in vivo.

### LINC00958 acts as a ceRNA via binding to miR-211-5p in TSCC

**Fig. 4:**
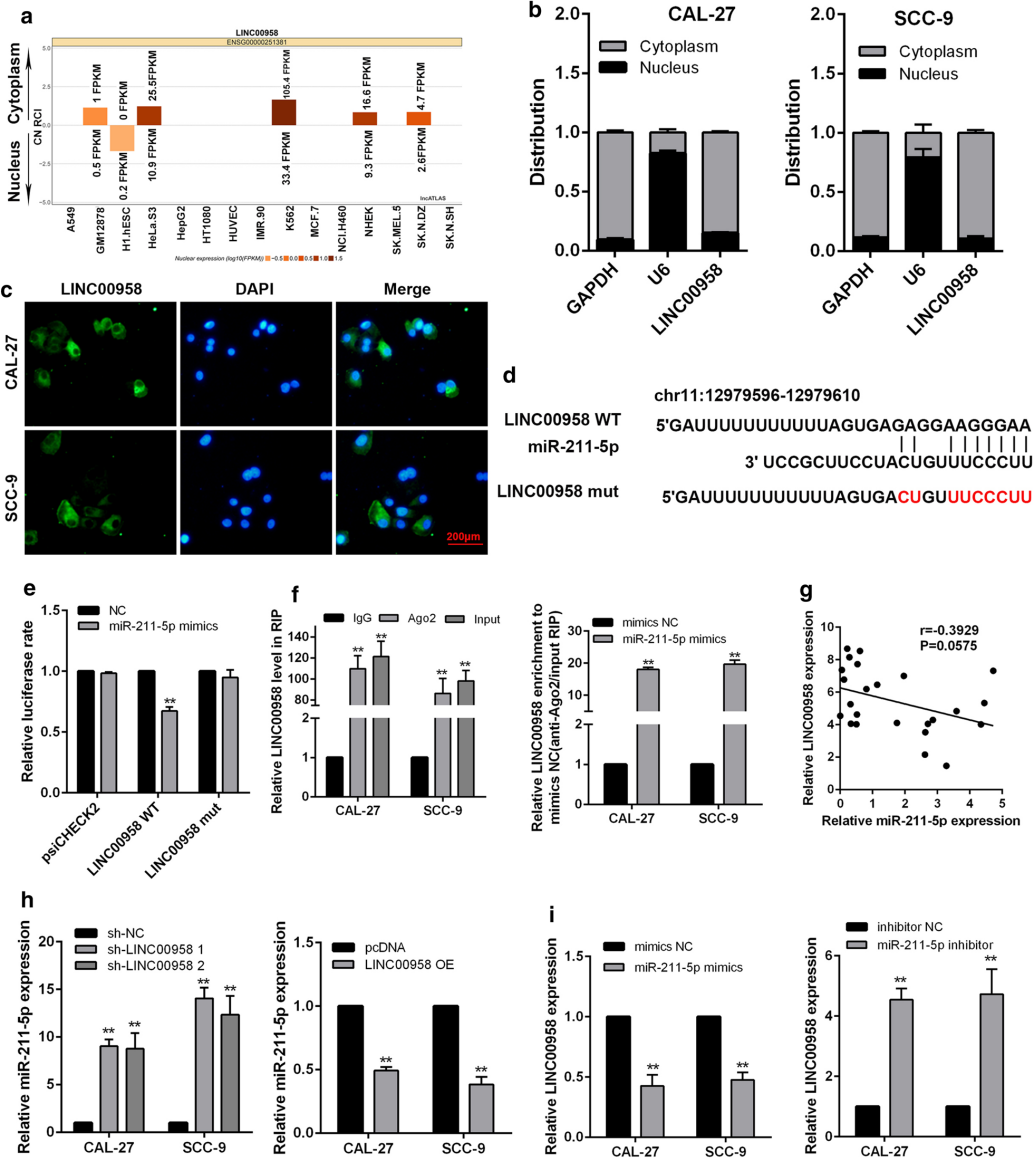
LINC00958 acts as a ceRNA via binding to miR-211-5p. **a** LINC00958 was predicted to be located mainly in the cytoplasm using the bioinformatics tools in lncATLAS. **b** RT-qPCR analysis of subcellular LINC00958 expression in the nucleus and cytoplasm of CAL-27 and SCC-9 cells. GAPDH, β-actin and U6 were used as endogenous controls. **c** FISH assay was performed to detect the subcellular localization of LINC00958 in CAL-27 and SCC-9 cells. LINC00958 is stained green and nuclei are stained blue (DAPI). **d** The predicted miR-211-5p binding sites in the LINC00958 transcript. **e** Luciferase activities in CAL-27 cells co-transfected wild-type (WT) or mutant LINC00958 plasmid together with miR-211-5p mimic or miR-Ctrl. **f** Enrichment of LINC00958 and miR-211-5p in the Ago2 immunoprecipitation compared with the control IgG precipitation. **g** Correlation between LINC00958 and miR-211-5p expression in TSCC tissues based on the TCGA database. **h** RT-qPCR of miR-211-5p in CAL-27 and SCC-9 cells stably transfected with sh-LINC00958, pcDNA-LINC00958 and their corresponding control vectors. **i** RT-qPCR of LINC00958 in CAL-27 and SCC-9 cells transfected with miR-211-5p mimics, miR-211-5p inhibitor and their corresponding controls ^*^P < 0.05, ^**^P < 0.01, ^***^P < 0.001

LINC00958 is highly expressed in various types of cancers and could act as a ceRNA[20, 21]. To explore if LINC00958 acts as a miRNA sponge that competes with mRNA, we first predicted its subcellular localization with lncATLAS (http://lncatlas.crg.eu/). LINC00958 was predicted to be located mainly in the cytoplasm in a variety of cell types (Fig. 4a). RT-qPCR and FISH assays verified that LINC00958 was localized mainly in the cytoplasm of TSCC cells (Fig. 4b, c). Using LncRNABase (www.lncrnadb.org) and NONCODE (www.noncode.org) online database, we found potential binding sites between LINC00958 and miR-211-5p (Fig. 4d). Based on this prediction, luciferase reporter constructs carrying LINC00958 reporter were generated. The luciferase reporter assays showed that miR-211-5p mimics significantly decreased the luciferase activity of the wild type (Wt) reporter, but not that of mutant (Mut) reporter (Fig. 4e), indicating that the binding sequences were synergistically responsible for the interaction of LINC00958 and miR-211-5p. To further test if LINC00958 regulated miR-211-5p by acting as a ceRNA, RIP assays were performed with TSCC cells extracts using anti-Ago2. As shown in Fig. 4f, LINC00958 and miR-211-5p were substantially enriched in the Ago2 immunoprecipitation compared with the negative control IgG. In addition, miR-211-5p significantly increased the enrichment of LINC00958 by Ago2 immunoprecipitation. We also found the expression of miR-211-5p was significantly decreased in TSCC samples compared with the adjacent normal tissues (Additional file 5: Figure S3A, B). And the expression of miR-211-5p correlated negatively with that of LINC00958 in the TSCC tissues (Fig. 4g, Additional file 5: Figure S3C). Further investigations showed that LINC00958 negatively affected miR-211-5p expression levels (Fig. 4h), whereas LINC0095 expression was obviously suppressed by overexpression of miR-211-5p and elevated by ihibition of miR-211-5p (Fig. 4i). All these results indicated that the modulating effect of LINC00958 on miR-211-5p expression may be possibly mediated by its ability to act as a ceRNA.

### MiR-211-5p binds to CENPK and represses its expression

**Fig. 5:**
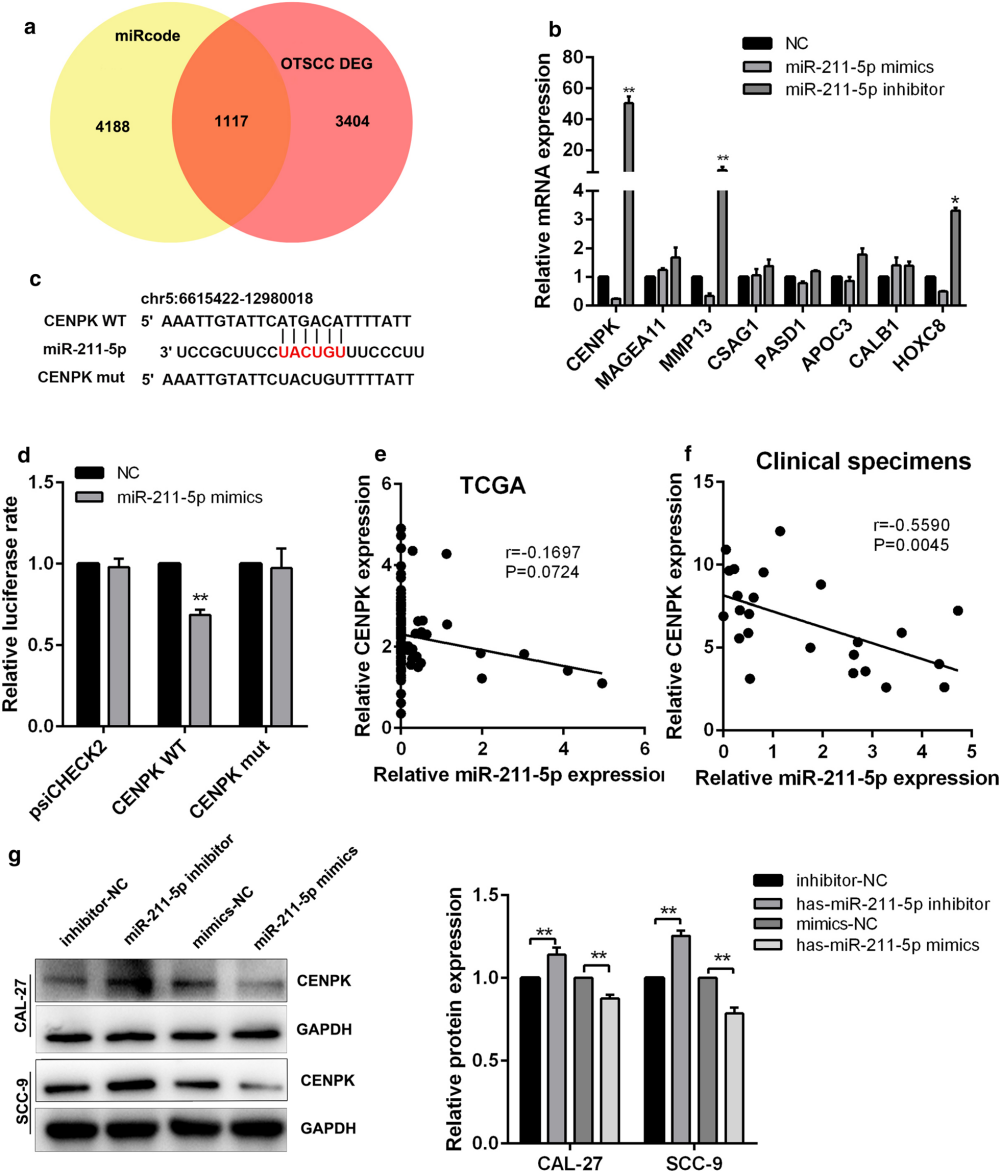
MiR-211-5p binds to CENPK and represses its expression. **a** The overlapping mRNAs from the online analysis tools miRcode (http://www.mircode.org/) and TCGA database. **b** RT-qPCR of potential target mRNAs of miR-211-5p in CAL-27 cells transfected with miR-211-5p mimics or miR-211-5p inhibitor. **c** Schematic diagram of the predicted binding sites between CENPK and miR-211-5p. **d** Luciferase activities in CAL-27 cells co-transfected wild-type (WT) or mutant CENPK plasmid together with miR-211-5p mimic or miR-Ctrl. **e** Correlation between CENPK and miR-211-5p expression in TSCC tissues based on the TCGA analysis. **f** Correlation between CENPK and miR-211-5p expression in TSCC clinical samples. **g** Relative protein level of CENPK in CAL-27 and SCC-9 cells transfected with miR-211-5p mimics, miR-211-5p inhibitor and their corresponding controls. ^*^P < 0.05, ^**^P < 0.01, ^***^P < 0.001

The preceding results suggested that LINC00958 regulates miR-211-5p possibly by acting as a ceRNA. Thus, we searched the possible targets of miR-211-5p by using a bioinformatics prediction tool—miRcode (http://www.mircode.org/) and analyzed the differentially expressed mRNAs in TCGA database between adjacent normal tissues and TSCC tissues (Fig. 5a). Among the common genes, eight of the most up-regulated mRNAs were subjected to validation by RT-qPCR analysis in CAL-27 cells with miR-211-5p inhibitor or miR-211-5p mimics administration. The results revealed that CENPK was the most notably up-regulated transcript in the miR-211-5p inhibitor group and down-regulated in the mimics group (Fig. 5b). The binding site between CENPK and miR-211-5p is shown in Fig. 5c. The results of luciferase reporter assays showed that the binding sequences were synergistically responsible for the interaction between CENPK and miR-211-5p (Fig. 5d). We also verified a negtive correlation bwtween CENPK and mir-211-5p based on the TSCC TCGA database and frozen clinical specimen (Fig. 5e, f). Furthermore, the expression of CENPK was significantly increased in TSCC samples compared with the adjacent normal tissues (Additional file 5: Figure S3D, E). Further investigations showed that miR-211-5p negatively regulated CENPK expression (Fig. 5g). Therefore, we confirmed that miR-211-5p suppressed CENPK expression in TSCC cells.

### LINC00958 promotes CENPK-mediated proliferation through miR-211-5p sponging in vitro

**Fig. 6:**
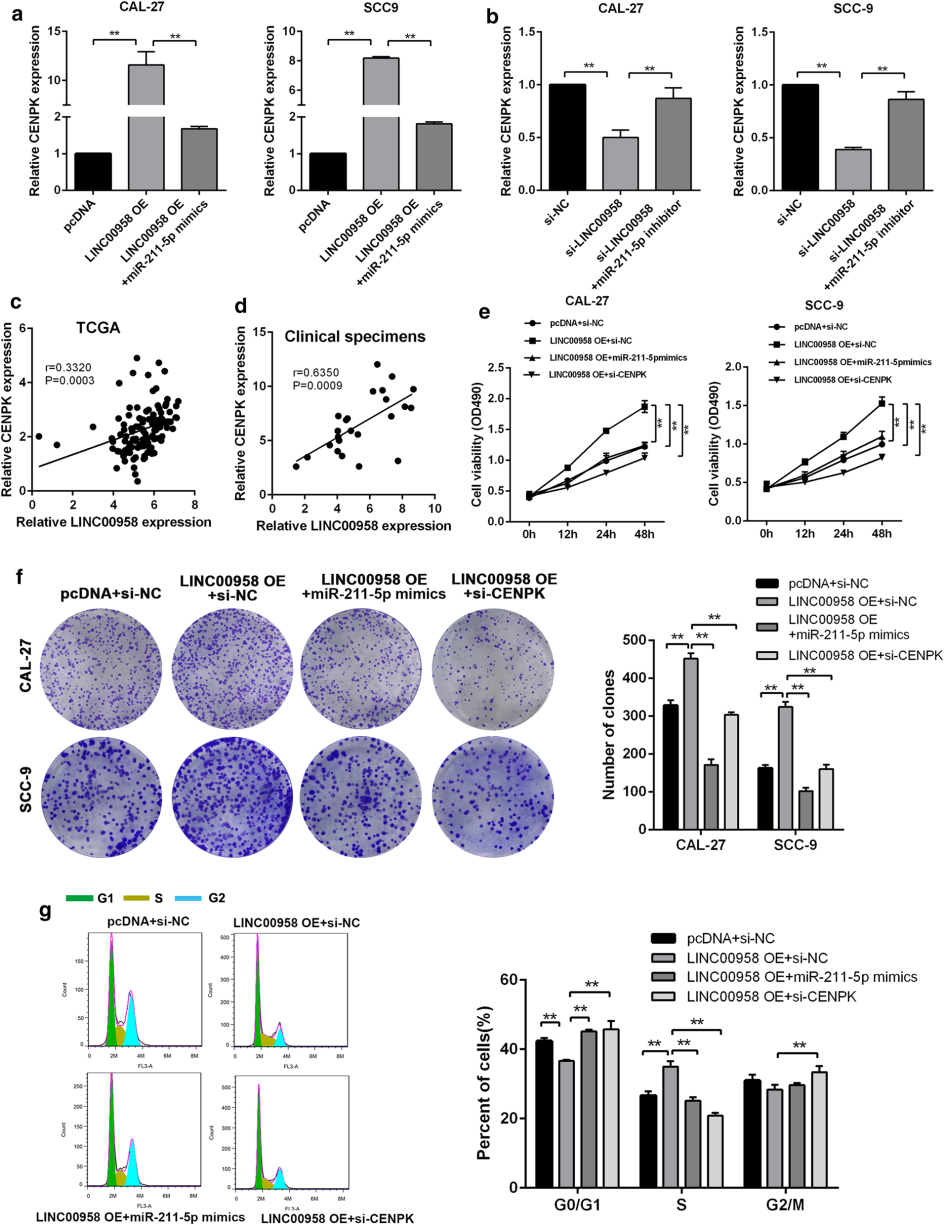
LINC00958 promotes CENPK mediated proliferation through miR-211-5p sponging in vitro. **a** RT-qPCR of CENPK in CAL27 and SCC-9 cells co-transfected with pcDNA-LINC00958 and miR-211-5p mimics. **b** RT-qPCR of CENPK in CAL27 and SCC-9 cells transfected with sh-LINC00958 or sh-NC simultaneously with miR-211-5p inhibitor. **c** Correlation between CENPK and LINC00958 expression in TSCC cells based on the TCGA database. **d** Correlation between CENPK and LINC00958 expression in TSCC frozen clinical samples. **e** CCK8 assays of CAL27 and SCC9 cells co-transfected with pcDNA-LINC00958 and miR-211-5p mimics or si-CENPK. **f** Colony formation assays of CAL27 and SCC9 cells co-transfected with pcDNA-LINC00958 and miR-211-5p mimics or si-CENPK. **g** Cell cycle distribution of CAL27 and SCC9 cells co-transfected with pcDNA-LINC00958 and miR-211-5p mimics or si-CENPK. ^*^P < 0.05, ^**^P < 0.01, ^***^P < 0.001

Next, we examed the effect of LINC00958 on CENPK. LINC00958 increased CENPK expression, which could be attenuated by miR-211-5p mimics (Fig. 6a). In addition, miR-211-5p inhibition successfully rescued the effect of LINC00958 silencing on CENPK expression (Fig. 6b). Pearson correlation analysis showed that the expression of CENPK positively correlated with that of LINC00958 in TSCC tissues (Fig. 6c, d). What’s more, the expression of miR-211-5p was dramatically increased in xenografts derived from LINC00958 knockdown group, while CENPK expression was decreased (Additional file 5: Figure S3F). To verify if LINC00958 modulate TSCC progression in a CENPK-dependent manner, we co-transfected CAL-27 and SCC-9 cells,with pcDNA-LINC00958 and si-CENPK or miR-211-5p mimics. CCK-8 assays, colony formation assays and Flow cytometry analysis demonstrated that the CENPK silencing or miR-211-5p overexpression weaken the promotion effects of LINC00958 over-expression on TSCC cell proliferation (Fig. 6e–g). These findings demonstrated that LINC00958 is an oncogenic lncRNA that promotes TSCC cell proliferation via the LINC00958/ miR-211-5p/ CENPK axis.

### LINC00958/mir-211-5p/CENPK axis promotes TSCC proliferation by regulating cell cycle process and activating the JAK/ STAT3 signaling pathway

**Fig. 7:**
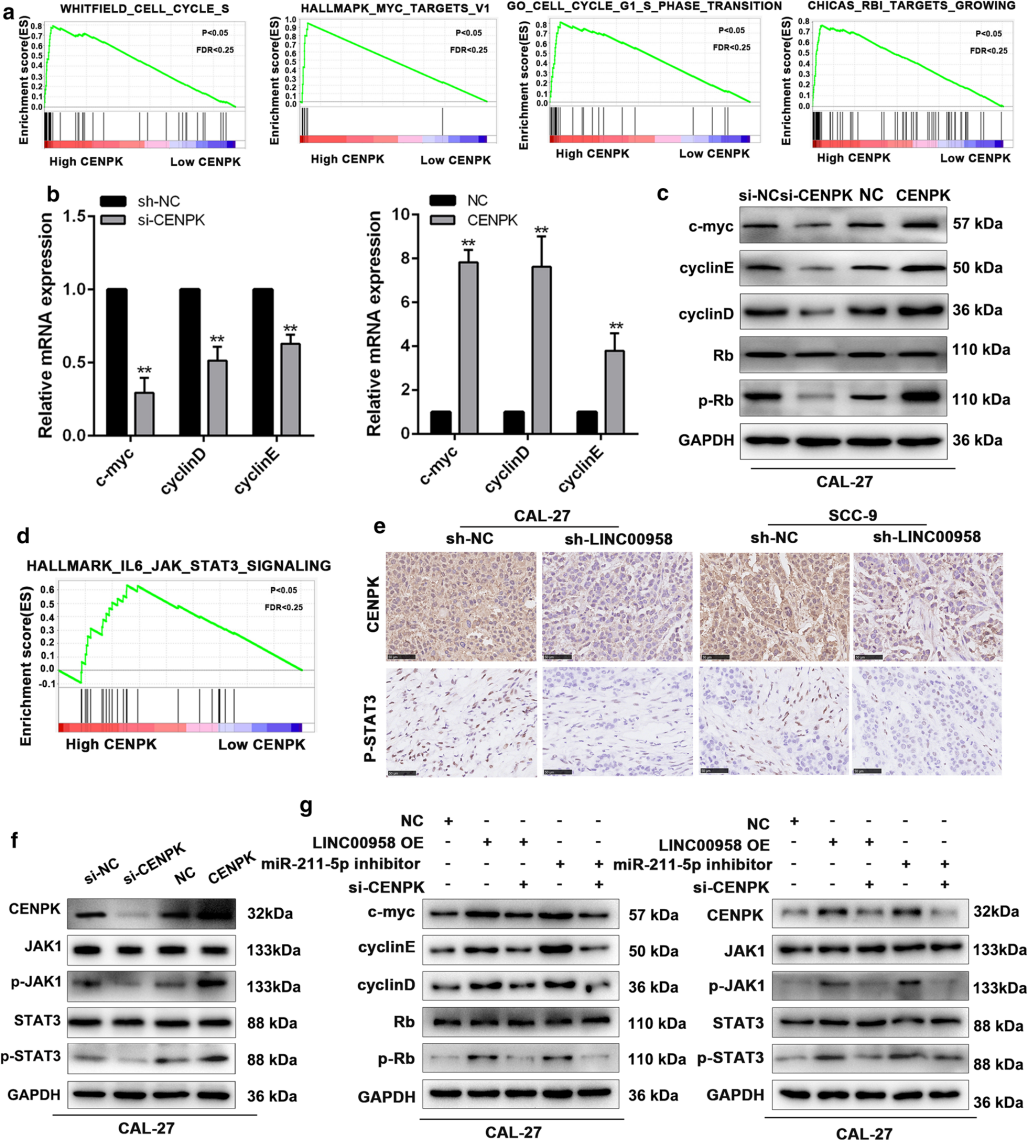
LINC00958/ mir-211-5p/ CENPK axis promotes TSCC proliferation via regulating cell-cycle process and activating the JAK/ STAT3 signaling pathway. **a** GSEA revealed that cell cycle related biological functions were enriched in response to high CENPK expression. **b** RT-qPCR of c-myc, cyclin D and cyclin E in CAL-27 cells overexpression or silencing CENPK. **c** The protein levels of c-myc, cyclin D, cyclin E, Rb and p-Rb in CAL-27 cells overexpression or silencing CENPK. **d** GSEA revealed that the JAK/STAT3 signaling pathway was enriched in response to high CENPK expression. **e** IHC of CENPK and p-STAT3 expression in the xenografts. **f** The protein levels of JAK1, p-JAK1, STAT3, p-STAT3 in CAL-27 cells overexpression or silencing CENPK. **g** The cell cycle related and JAK/STAT3 signaling related protein levels in CAL-27 cells co-transfected with pcDNA-LINC00958 and miR-211-5p mimics or si-CENPK. ^*^P < 0.05, ^**^P < 0.01, ^***^P < 0.001

**Fig. 8:**
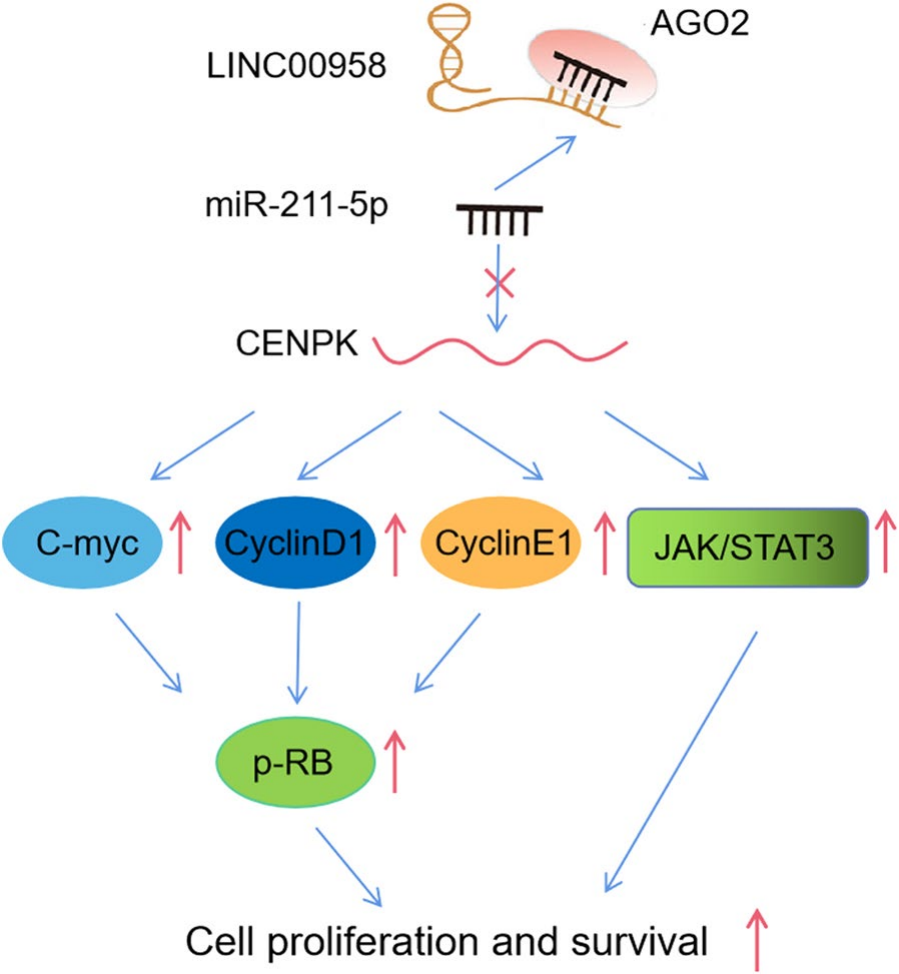
Proposed model of the mechanism underlying the expression and function of LINC00958 in TSCC cell proliferation

To better understand the mechanism by which CENPK regulates cell proliferation in TSCC, we referred to gene set enrichment analysis (GSEA) and found that CENPK expression was related to c-MYC target and cell cycle function. (Fig. 7a, Additional file 6: Figure S4A). CENPK over-expression increased the levels of c-myc, cyclinD, cyclinE, and p-RB, whereas the knockdown of CENPK resulted in the opposite effects (Fig. 7b, c). Furthermore, GSEA analysis suggested that CENPK might influence the JAK/STAT3 signaling pathway (Fig. 7d and Additional file 7: Table S3). IHC analysis showed a decreased CENPK and p-STAT3 levels in LINC00958 silenced xenografts (Fig. 7e).Western blot assays further verified that the overexpression of CENPK positively affected the expression of p-JAK1 and p-STAT3 at the protein levels (Fig. 7f). Moreover, LINC00958 could also elevated the above protein levels, while CENPK silencing or miR-211-5p overexpression could restain these promotion effects (Fig. 7g).Together, these findings demonstrated that LINC00958-induced CENPK promotes TSCC tumorigenesis through the modulation of the cell cycle process and the activation of the JAK/STAT3 signaling pathway (Fig. 8).

## Discussion

Accumulating evidence indicates that lncRNAs dysregulation plays important roles in tumorigenesis and in the progression of various types of cancers, which provides new perspectives for the treatment of malignant cancers. Some reports have pointed out that some lncRNAs serve as a scaffold of protein complex to promote or inhibit gene expression at the transcription level [22], but lncRNAs can also act as a mediator in the ceRNA pathway to regulate tumorigenesis [23]. LINC00958 has been identified as an oncogene in human cancers. It could bind with miR-330-5p, and silencing LINC00958 could inhibit EMT, tumor growth, invasion and metastasis of prostate cancer cells [21]. LINC00958 could promote lymph node metastasis by inducing tumor-associated lymphangiogenesis and promoting bladder cancer cell invasiveness [24]. In addition, cell proliferation could be suppressed and apoptosis could be elevated by combining radiotherapy with LINC00958 silencing in cervical cancer cells [20]. Moreover, overexpression of long non-coding facilitated cell proliferation, migration and invasion in lung adenocarcinoma [25]. In the present study, LINC00958 was also up-regulated in TSCC tissues when compared with pair matched normal tissues, and the upregulation of LINC00958 was associated with clinical characteristics and poor overall survival. We also determined the effects of LINC00958 on TSCC cells. The inhibition of LINC00958 suppressed the proliferation of TSCC cells in vitro and in vivo, and LINC00958 promoted TSCC cell proliferation by sponging miR-211-5p, leading to enhanced CENPK expression and the activation of the JAK/STAT3 signaling pathway. Collectively, our results indicated that LINC00958, acting as an oncogene, may be a potential diagnostic and prognostic biomarker, as well as a therapeutic target in TSCC.

Growing evidence supports the existence of a novel and extensive interaction network involving ceRNAs [26, 27], in which lncRNAs regulate miRNAs by competitively binding their target sites on protein-coding mRNA molecules. To a large extent, the level of regulation depends on the cellular location of the lncRNA [28]. LncRNAs located in the cytoplasm always function as endogenous miRNA sponges for miRNA response elements, thereby impairing the function of target mRNA at the posttranscriptional level [29]. In the present study, using FISH assay and RT-qPCR detection of RNAs in the cell cytoplasm or nucleus, we noted that LINC00958 is a cytoplasmic long noncoding RNA, which suggested that LINC00958 might exert its function as a ceRNA at the post-transcription level. Thus, to verify the ceRNA mechanism of LINC00958, we searched for candidate miRNAs.

A large number of lncRNAs have shown the capacity to act as sponges for miRNA and exert functions in tumorigenesis and tumor progression. For example, LINC01234 acts as a ceRNA to regulate CBFB expression by sponging miR-204-5p to promote gastric cancer cell proliferation [30]. UICLM promoted colorectal cancer metastasis by acting as a ceRNA which sponges miRNA-215 to regulate ZEB2 expression [31]. In this study, using bioinformatics tools as well as dual luciferase reporter and RNA pull-down assays, we showed that miR-211-5p directly bound to LINC00958. In addition, the expression of miR-211-5p was inversely related to that of LINC00958 in TSCC samples. Knockdown of LINC00958 promoted the expression of miR-211-5p. As previously reported in a mount of researches, lncRNA could binds with miRNA in MREs and downregulate its expression when working as a ceRNA. The mechanism for how lncRNA decrease miRNA was not fullly understand, one way through which a relatively lncRNA can nevertheless regulate the activity of typically miRNAs is through binding sites with special sequences or pairing topology, that would trigger miRNA degradation upon binding. In detail, binding of miRNAs loaded in Ago1 to targets with extensive sequence complementarity triggers tailing of the miRNA with non-templated nucleotides (mostly adenines and uridines), miRNA trimming, and eventual miRNA degradation a phenomenon referred to as target RNA‐directed miRNA degradation or TDMD [32].

MiRNAs directly bind to the 3′-UTR of downstream targeting genes involved in tumor progression [33]. MiR-211-5p-mediated inhibition of SNAIL1 expression contributes to the suppression of RCC progression [18]. Moreover, miR-211-5p overexpression suppressed the proliferation, migration, and invasion of triple-negative breast cancer [34]. Using an online database, we predicted CENPK as a potential target of miR-211-5p, which was confirmed by luciferase reporter and RT-qPCR assays. CENPK is known to be a subunit of the CENPH-I complex, which is essential for proper kinetochore assembly [35]. Several studies have found that CENPK is overexpressed in several tumor types and promotes tumor progression. In the present study, our results showed that CENPK was up-regulated in clinical samples. In addition, the expression of miR-211-5p was inversely related to that of CENPK in TSCC samples. The overexpression of miR-211-5p inhibited CENPK protein expression. Further investigation found that the expression of miR-211-5p was negatively associated with that of CENPK in TSCC samples. The overexpression of CENPK promoted the expression of proliferation-related protein, such as c-myc, cyclin D, cyclin E and p-Rb. It demonstrated that LINC00958 up-regulated CENPK expression by competitively sponging miR-211-5p, thus promoting TSCC cell proliferation.

Signal transducer and activator of transcription 3 plays a crucial role in a wide variety of biological processes such as cell proliferation, invasion, apoptosis and immunity [36]. Numerous studies have reported that STAT3 activation is associated with a poor prognosis of diverse cancers including, colorectal cancer [37] and hepatocellular carcinoma [38]. In addition, lncRNA ATB activated AKT and the JAK/STAT3 signaling pathway through down-regulated miR-494 in lung cancer [39]. However, the molecular mechanisms responsible for the activation of JAK/STAT3 signaling pathway in TSCC are still poorly understood. The data from GSEA demonstrated that JAK/STAT3 signaling pathway related-genes are enriched in TSCC patients expressing high level of CENPK. Our study dissected the mechanisms by which CENPK mediates the JAK/STAT3 signaling pathway activation. The overexpression of CENPK promoted the expression of p-JAK1 and p-STAT3. In addition, we also demonstrated that LINC00958 knockdown decreased the expression of CENPK and p-STAT3 in vivo. Thus, these findings uncovered the novel mechanism associated with the activation of STAT3 in TSCC.

## Conclusions

In conclusion, we identified LINC00958 as an oncogenic lncRNA in TSCC. Functional and mechanistic analysis revealed that LINC00958 promotes TSCC cell proliferation by acting as a ceRNA which sponges miR-211-5p, causing enhanced CENPK expression and the activation of the JAK/STAT3 signaling pathway. Our study demonstrates that LINC00958 plays an important role in TSCC tumorigenesis and progression. Furthermore, it elucidates the clinical significance and regulatory mechanism of LINC00958 in TSCC and provides a prognostic indicator as well as a promising therapeutic target for TSCC patients.

## Supplementary Information

**Additional file 1. Table S1.** Correlation of clinicopathological characteristics and gene expression in the patients.

**Additional file 2. Figure S1.** (A) Hierarchical clustering analysis the differentially expressed lncRNAs in TSCC tumor tissues from TCGA database. (B)The expression of different expressed lncRNAs were subject to validation by RT-qPCR. (C) Kaplan-Meier analysis of the correlation between LINC00958 expression and overall survival in different tumors. *P<0.05, **P<0.01, ***P<0.001.

**Additional file 3. Table S2.** Sequences of PCR primers used in this study.

**Additional file 4. Figure S2.** (A) RT-qPCR analysis of LINC00958 in CAL-27 (A) and SCC-9 (B) cells stably transfected with sh-LINC00958, pcDNA-LINC00958 and their corresponding control vectors. (B) EDU assay of CAL27 and SCC9 cells were stably transfected with sh-LINC00958, pcDNA-LINC00958 and their corresponding control vectors. ^*^P<0.05, ^**^P<0.01, ^***^P<0.001.

**Additional file 5. Figure S3.** (A) The expression of miR-211-5p in TSCC tissues and normal tissues based on the TCGA database. (B) RT-qPCR of miR-211-5p in TSCC tissues (n=24) and normal tissues (n=24) (C) Correlation between LINC00958 and miR-211-5p expression in TSCC TCGA database. (D) The expression of CENPK in TSCC tissues and normal tissues based on TCGA database. (E) The expression of CENPK in TSCC tissues and normal tissues based on TCGA database. (F) RT-qPCR of miR-211-5p and CENPK in xenografts. *P<0.05, **P<0.01, ***P<0.001.

**Additional file 6. Figure S4.** (A) TCGA analysis revealed that cell cycle-related biological functions were enriched in response to high CENPK expression. (B) The protein levels of c-myc, cyclin D, cyclin E, Rb and p-Rb in SCC-9 cells overexpression or silencing CENPK. (C) The protein levels of JAK1, p-JAK1, STAT3, p-STAT3 in SCC-9 cells overexpression or silencing CENPK.

**Additional file 7. Table S3.** HALLMARK_IL6_ JAK_STAT3_SIGNALING Gene Set Enrichment.

## Data Availability

All data are fully available without restrictions.

## Abbreviations

lncRNAs: Long non-coding RNAs
LINC00958: Long intergenic noncoding RNA 00958
RT-qPCR: Quantitative real-time polymerase chain reaction
EdU: 5-Ethynyl-2′-deoxyuridline
CENPK: Centromere protein K
TSCC: Tongue squamous cell carcinoma
IHC: Immunohistochemistry
ceRNA: Competing endogenous RNA
shRNA: Short hairpin RNA
SMC: Smooth muscle cell
GAPDH: Glyceraldehyde-3-phosphate dehydrogenase
PVDF: Polyvinylidene fluoride

## References

[1] Chen W, Zheng R, Baade PD, Zhang S, Zeng H, Bray F, Jemal A, Yu XQ, He J (2016). Cancer statistics in China, 2015. CA Cancer J Clin.

[2] Wang ZY, Hu M, Dai MH, Xiong J, Zhang S, Wu HJ, Zhang SS, Gong ZJ (2018). Upregulation of the long non-coding RNA AFAP1-AS1 affects the proliferation, invasion and survival of tongue squamous cell carcinoma via the Wnt/beta-catenin signaling pathway. Mol Cancer.

[3] Tang Q, Cheng B, Xie M, Chen Y, Zhao J, Zhou X, Chen L (2017). Circadian Clock Gene Bmal1 inhibits tumorigenesis and increases paclitaxel sensitivity in tongue squamous cell carcinoma. Cancer Res.

[4] Chen WC, Li QL, Pan Q, Zhang HY, Fu XY, Yao F, Wang JN, Yang AK (2019). Xenotropic and polytropic retrovirus receptor 1 (XPR1) promotes progression of tongue squamous cell carcinoma (TSCC) via activation of NF-kappaB signaling. J Exp Clin Cancer Res.

[5] Siegel R, Ward E, Brawley O, Jemal A (2011). Cancer statistics, 2011: the impact of eliminating socioeconomic and racial disparities on premature cancer deaths. CA Cancer J Clin.

[6] Karatas OF, Oner M, Abay A, Diyapoglu A (2017). MicroRNAs in human tongue squamous cell carcinoma: from pathogenesis to therapeutic implications. Oral Oncol.

[7] Huarte M (2015). The emerging role of lncRNAs in cancer. Nat Med.

[8] Mercer TR, Dinger ME, Mattick JS (2009). Long non-coding RNAs: insights into functions. Nat Rev Genet.

[9] Xu Z, Yang F, Wei D, Liu B, Chen C, Bao Y, Wu Z, Wu D, Tan H, Li J (2017). Long noncoding RNA-SRLR elicits intrinsic sorafenib resistance via evoking IL-6/STAT3 axis in renal cell carcinoma. Oncogene.

[10] Yang X, Cai JB, Peng R, Wei CY, Lu JC, Gao C, Shen ZZ, Zhang PF, Huang XY, Ke AW (2019). The long noncoding RNA NORAD enhances the TGF-beta pathway to promote hepatocellular carcinoma progression by targeting miR-202-5p. J Cell Physiol.

[11] Kou N, Liu S, Li X, Li W, Zhong W, Gui L, Chai S, Ren X, Na R, Zeng T (2019). H19 facilitates tongue squamous cell carcinoma migration and invasion via sponging miR-let-7. Oncol Res.

[12] Zhang S, Ma H, Zhang D, Xie S, Wang W, Li Q, Lin Z, Wang Y (2018). LncRNA KCNQ1OT1 regulates proliferation and cisplatin resistance in tongue cancer via miR-211-5p mediated Ezrin/Fak/Src signaling. Cell Death Dis.

[13] Wang Z, Zhu X, Dong P, Cai J (2020). Long noncoding RNA LINC00958 promotes the oral squamous cell carcinoma by sponging miR-185-5p/YWHAZ. Life Sci.

[14] Huang S, Zhan Z, Li L, Guo H, Yao Y, Feng M, Deng J, Xiong J (2019). LINC00958-MYC positive feedback loop modulates resistance of head and neck squamous cell carcinoma cells to chemo- and radiotherapy in vitro. Onco Targets Ther.

[15] Filipowicz W, Bhattacharyya SN, Sonenberg N (2008). Mechanisms of post-transcriptional regulation by microRNAs: are the answers in sight?. Nat Rev Genet.

[16] Chen H, Zhang Z, Lu Y, Song K, Liu X, Xia F, Sun W (2017). Downregulation of ULK1 by microRNA-372 inhibits the survival of human pancreatic adenocarcinoma cells. Cancer Sci.

[17] Yan J, Jia Y, Chen H, Chen W, Zhou X (2019). Long non-coding RNA PXN-AS1 suppresses pancreatic cancer progression by acting as a competing endogenous RNA of miR-3064 to upregulate PIP4K2B expression. J Exp Clin Cancer Res.

[18] Wang K, Jin W, Jin P, Fei X, Wang X, Chen X (2017). miR-211-5p suppresses metastatic behavior by targeting SNAI1 in renal cancer. Mol Cancer Res.

[19] Jiang G, Wen L, Deng W, Jian Z, Zheng H (2017). Regulatory role of miR-211-5p in hepatocellular carcinoma metastasis by targeting ZEB2. Biomed Pharmacother.

[20] Zhao H, Zheng GH, Li GC, Xin L, Wang YS, Chen Y, Zheng XM (2019). Long noncoding RNA LINC00958 regulates cell sensitivity to radiotherapy through RRM2 by binding to microRNA-5095 in cervical cancer. J Cell Physiol.

[21] Chen S, Chen JZ, Zhang JQ, Chen HX, Qiu FN, Yan ML, Tian YF, Peng CH, Shen BY, Chen YL (2019). Silencing of long noncoding RNA LINC00958 prevents tumor initiation of pancreatic cancer by acting as a sponge of microRNA-330-5p to down-regulate PAX8. Cancer Lett.

[22] Tsai MC, Manor O, Wan Y, Mosammaparast N, Wang JK, Lan F, Shi Y, Segal E, Chang HY (2010). Long noncoding RNA as modular scaffold of histone modification complexes. Science.

[23] Zheng ZQ, Li ZX, Zhou GQ, Lin L, Zhang LL, Lv JW, Huang XD, Liu RQ, Chen F, He XJ (2019). Long noncoding RNA FAM225A promotes nasopharyngeal carcinoma tumorigenesis and metastasis by acting as ceRNA to sponge miR-590-3p/miR-1275 and upregulate ITGB3. Cancer Res.

[24] He W, Zhong G, Jiang N, Wang B, Fan X, Chen C, Chen X, Huang J, Lin T (2018). Long noncoding RNA BLACAT2 promotes bladder cancer-associated lymphangiogenesis and lymphatic metastasis. J Clin Invest.

[25] Yang L, Li L, Zhou Z, Liu Y, Sun J, Zhang X, Pan H, Liu S (2020). SP1 induced long non-coding RNA LINC00958 overexpression facilitate cell proliferation, migration and invasion in lung adenocarcinoma via mediating miR-625-5p/CPSF7 axis. Cancer Cell Int.

[26] Thomson DW, Dinger ME (2016). Endogenous microRNA sponges: evidence and controversy. Nat Rev Genet.

[27] Yu C, Li L, Xie F, Guo S, Liu F, Dong N, Wang Y (2018). LncRNA TUG1 sponges miR-204-5p to promote osteoblast differentiation through upregulating Runx2 in aortic valve calcification. Cardiovasc Res.

[28] Yoon JH, Abdelmohsen K, Gorospe M (2013). Posttranscriptional gene regulation by long noncoding RNA. J Mol Biol.

[29] Tay Y, Rinn J, Pandolfi PP (2014). The multilayered complexity of ceRNA crosstalk and competition. Nature.

[30] Chen X, Chen Z, Yu S, Nie F, Yan S, Ma P, Chen Q, Wei C, Fu H, Xu T (2018). Long noncoding RNA LINC01234 functions as a competing endogenous RNA to regulate CBFB expression by sponging miR-204-5p in gastric cancer. Clin Cancer Res.

[31] Chen DL, Lu YX, Zhang JX, Wei XL, Wang F, Zeng ZL, Pan ZZ, Yuan YF, Wang FH, Pelicano H (2017). Long non-coding RNA UICLM promotes colorectal cancer liver metastasis by acting as a ceRNA for microRNA-215 to regulate ZEB2 expression. Theranostics.

[32] Ulitsky I (2018). Interactions between short and long noncoding RNAs. FEBS Lett.

[33] Min A, Zhu C, Peng S, Rajthala S, Costea DE, Sapkota D (2015). MicroRNAs as important players and biomarkers in oral carcinogenesis. Biomed Res Int.

[34] Chen LL, Zhang ZJ, Yi ZB, Li JJ (2017). MicroRNA-211-5p suppresses tumour cell proliferation, invasion, migration and metastasis in triple-negative breast cancer by directly targeting SETBP1. Br J Cancer.

[35] Cheeseman IM, Hori T, Fukagawa T, Desai A (2008). KNL1 and the CENP-H/I/K complex coordinately direct kinetochore assembly in vertebrates. Mol Biol Cell.

[36] Yu H, Pardoll D, Jove R (2009). STATs in cancer inflammation and immunity: a leading role for STAT3. Nat Rev Cancer.

[37] Liang Q, Ma D, Zhu X, Wang Z, Sun TT, Shen C, Yan T, Tian X, Yu T, Guo F (2018). RING-Finger protein 6 amplification activates JAK/STAT3 pathway by modifying SHP-1 ubiquitylation and associates with poor outcome in colorectal cancer. Clin Cancer Res.

[38] Jiang C, Long J, Liu B, Xu M, Wang W, Xie X, Wang X, Kuang M (2017). miR-500a-3p promotes cancer stem cells properties via STAT3 pathway in human hepatocellular carcinoma. J Exp Clin Cancer Res.

[39] Cao Y, Luo X, Ding X, Cui S, Guo C (2018). LncRNA ATB promotes proliferation and metastasis in A549 cells by down-regulation of microRNA-494. J Cell Biochem.

