# Supplementary material for: LINC00958 promotes the proliferation of TSCC via miR-211-5p/CENPK axis and activating the JAK/STAT3 signaling pathway
Source: Cancer Cell Int. 2021 Mar 3;21:147. doi: 10.1186/s12935-021-01808-z (PMC7931557; doi:10.1186/s12935-021-01808-z)
Supplement: Supplementary file 1 — Additional file 1. Table S1. Correlation of clinicopathological characteristics and gene expression in the patients. [file 12935_2021_1808_MOESM1_ESM.docx]

**Table S1. Sequences of PCR primers used in this study**

| HOXA10-AS | Forward(5’-3’) | CTGCGGAGAAAGACACGAG |
| --- | --- | --- |
|  | Reverse(5’-3’) | GCACGACACTGCCTTCATTAC |
| AP002957.1 | Forward(5’-3’) | CCTCACAACAAGCATTGAGACA |
|  | Reverse(5’-3’) | CGGGAAAGGGAGTGGATT |
| MIR4713HG | Forward(5’-3’) | ACACGGTGTGCGCACCCACTGACCT |
|  | Reverse(5’-3’) | AGGTCAGTGGGTGCGCACACCGTGT |
| FOXD2-AS1 | Forward(5’-3’) | GGTTGAGGGTCTGTGACTGTAG |
|  | Reverse(5’-3’) | CGGCGTGTAATTGGTAGGA |
| LINC00958 | Forward(5’-3’) | GCAGCAAGATAGCTCCAGGT |
|  | Reverse(5’-3’) | GCTCCGGCTGCTGAAAGATA |
| AC011611.3 | Forward(5’-3’) | TTCAGGCAGAGTTGGAGGTG |
|  | Reverse(5’-3’) | GGCAAGGAGATCGACTTTCG |
| CTD-2194D22.4 | Forward(5’-3’) | GGGACCATCACTTTGACTCTTA |
|  | Reverse(5’-3’) | CGGGTGCCCACTTCTTCT |
| CENPK | Forward(5’-3’) | CTTGAGTACCTTGGGCGAGT |
|  | Reverse(5’-3’) | GGCAATTCCATTACGCAGCA |
| MAGEA11 | Forward(5’-3’) | TGAGCAAGGTGAGCACTATGT |
|  | Reverse(5’-3’) | CCCACAGCACTTGTTCTCCT |
| MMP13 | Forward(5’-3’) | GTTCGGCCACTCCTTAGGTC |
|  | Reverse(5’-3’) | AAGTGGCTTTTGCCGGTGTA |
| PASD1 | Forward(5’-3’) | ATCTGTGCCCCTCTGCAATC |
|  | Reverse(5’-3’) | GGTCTCCAGGGTGCTTATGG |
| APOC3 | Forward(5’-3’) | GTTACATGAAGCACGCCACC |
|  | Reverse(5’-3’) | CACGGCTGAAGTTGGTCTGA |
| CALB1 | Forward(5’-3’) | GACGCTGACGGAAGTGGTTA |
|  | Reverse(5’-3’) | GAGCCAACAATCCAGCCTTCT |
| HOXC8 | Forward(5’-3’) | GCTCCTACTTCGTCAACCCC |
|  | Reverse(5’-3’) | AACCGGCAGTCGTAATAGGC |
| C-MYC | Forward(5’-3’) | CGTCCTCGGATTCTCTGCTC |
|  | Reverse(5’-3’) | GCTGCGTAGTTGTGCTGATG |
| cyclin D | Forward(5’-3’) | GATGCCAACCTCCTCAACGA |
|  | Reverse(5’-3’) | GGAAGCGGTCCAGGTAGTTC |
| CSAG1 | Forward(5’-3’) | CCTGCCTTCACTGTCCTG |
|  | Reverse(5’-3’) | CCTTCTGGGTTGTCTTG |
| cyclin E | Forward(5’-3’) | ACCAGTTTGCGTATGTGA |
|  | Reverse(5’-3’) | TGTGGGTCTGTATGTTGTG |
| miR-211-5p | Forward(5’-3’) | TTCCCTTTGTCATCCTTCGCCT |
