# Supplementary figures and images for: LINC00958 promotes the proliferation of TSCC via miR-211-5p/CENPK axis and activating the JAK/STAT3 signaling pathway
Source: Cancer Cell Int. 2021 Mar 3;21:147. doi: 10.1186/s12935-021-01808-z (PMC7931557; doi:10.1186/s12935-021-01808-z)

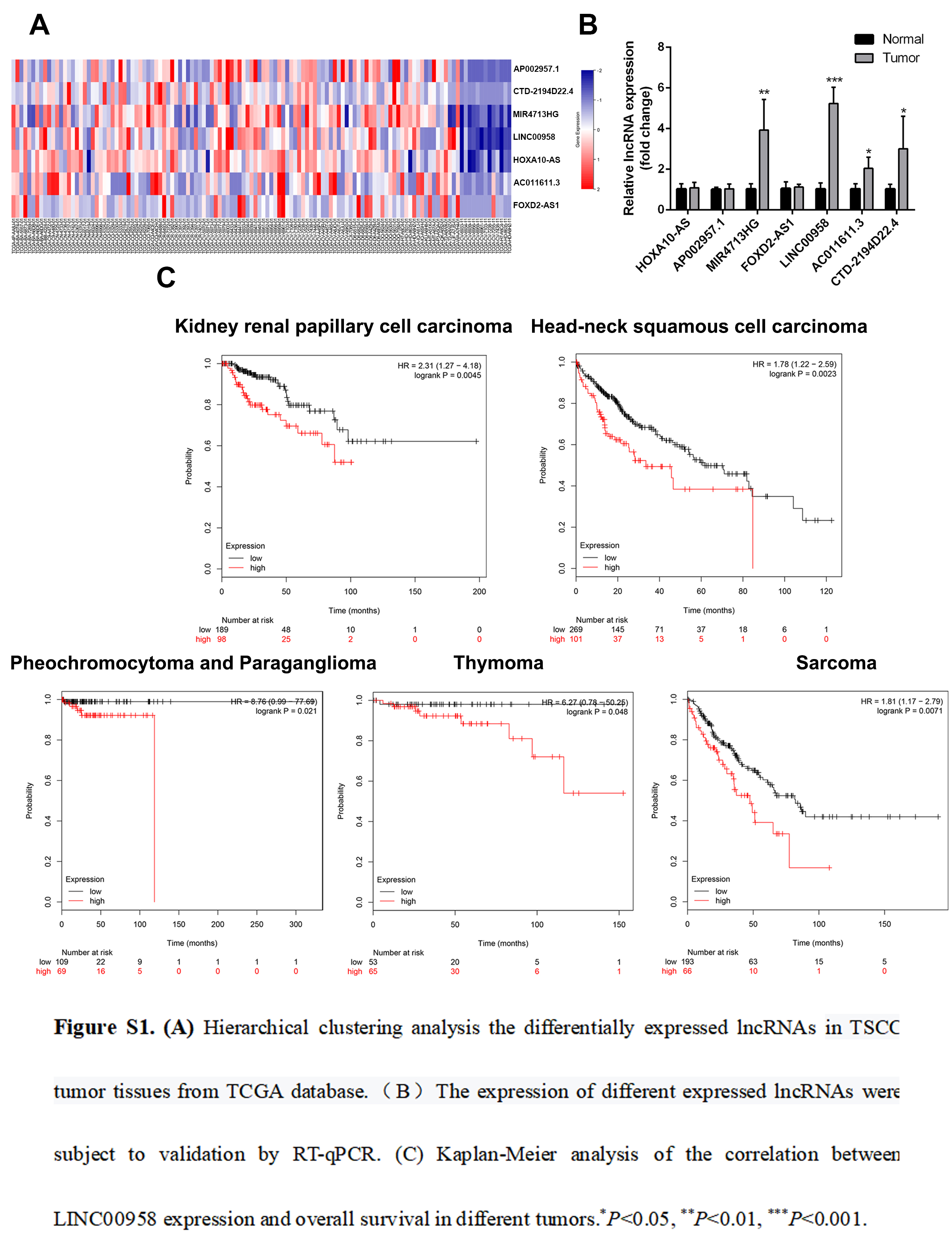

Supplement: Supplementary file 2 — Additional file 2. Figure S1. (A) Hierarchical clustering analysis the differentially expressed lncRNAs in TSCC tumor tissues from TCGA database. (B)The expression of different expressed lncRNAs were subject to validation by RT-qPCR. (C) Kaplan-Meier analysis of the correlation between LINC00958 expression and overall survival in different tumors. *P<0.05, **P<0.01, ***P<0.001. [file 12935_2021_1808_MOESM2_ESM.jpg]

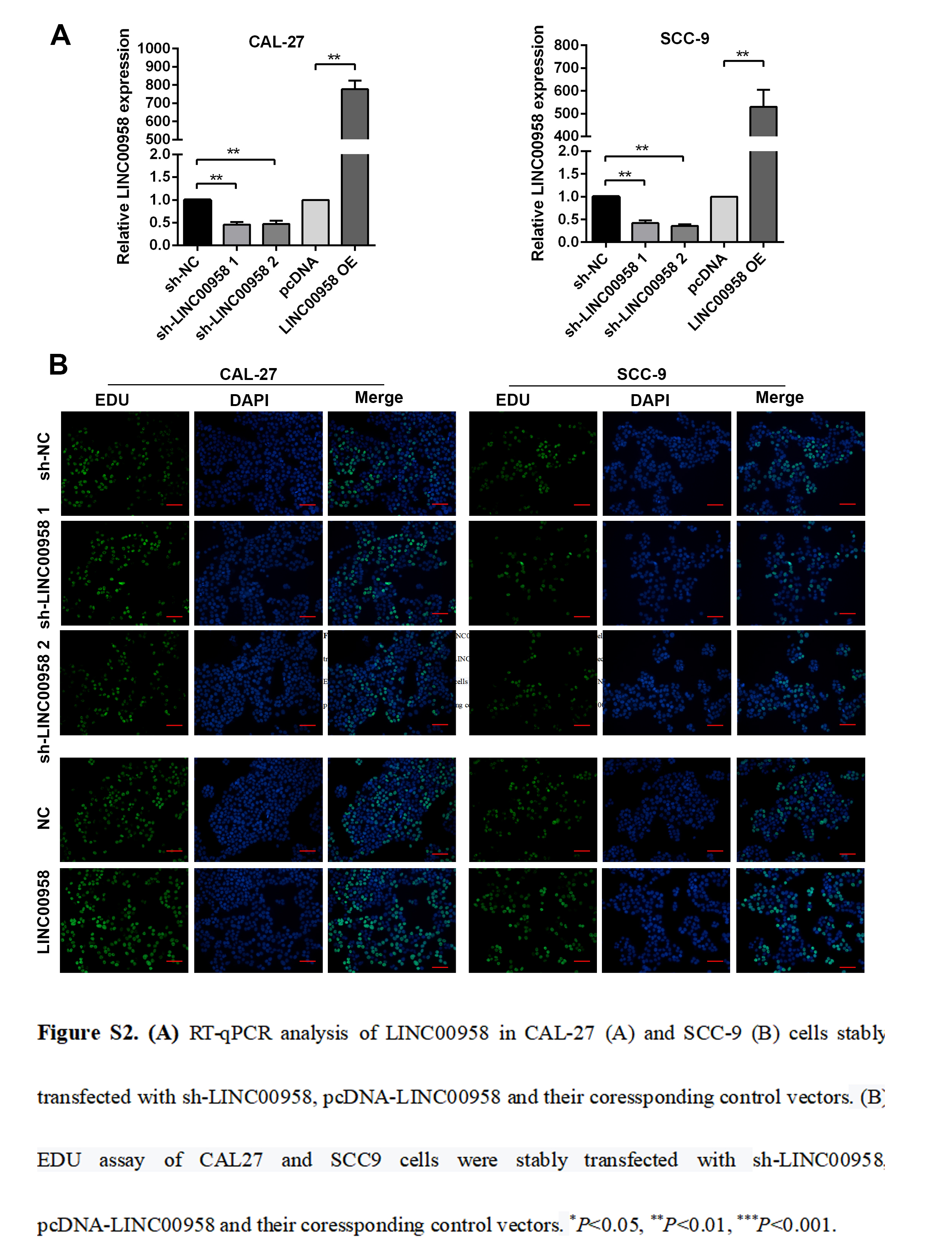

Supplement: Supplementary file 4 — Additional file 4. Figure S2. (A) RT-qPCR analysis of LINC00958 in CAL-27 (A) and SCC-9 (B) cells stably transfected with sh-LINC00958, pcDNA-LINC00958 and their corresponding control vectors. (B) EDU assay of CAL27 and SCC9 cells were stably transfected with sh-LINC00958, pcDNA-LINC00958 and their corresponding control vectors. *P<0.05, **P<0.01, ***P<0.001. [file 12935_2021_1808_MOESM4_ESM.jpg]

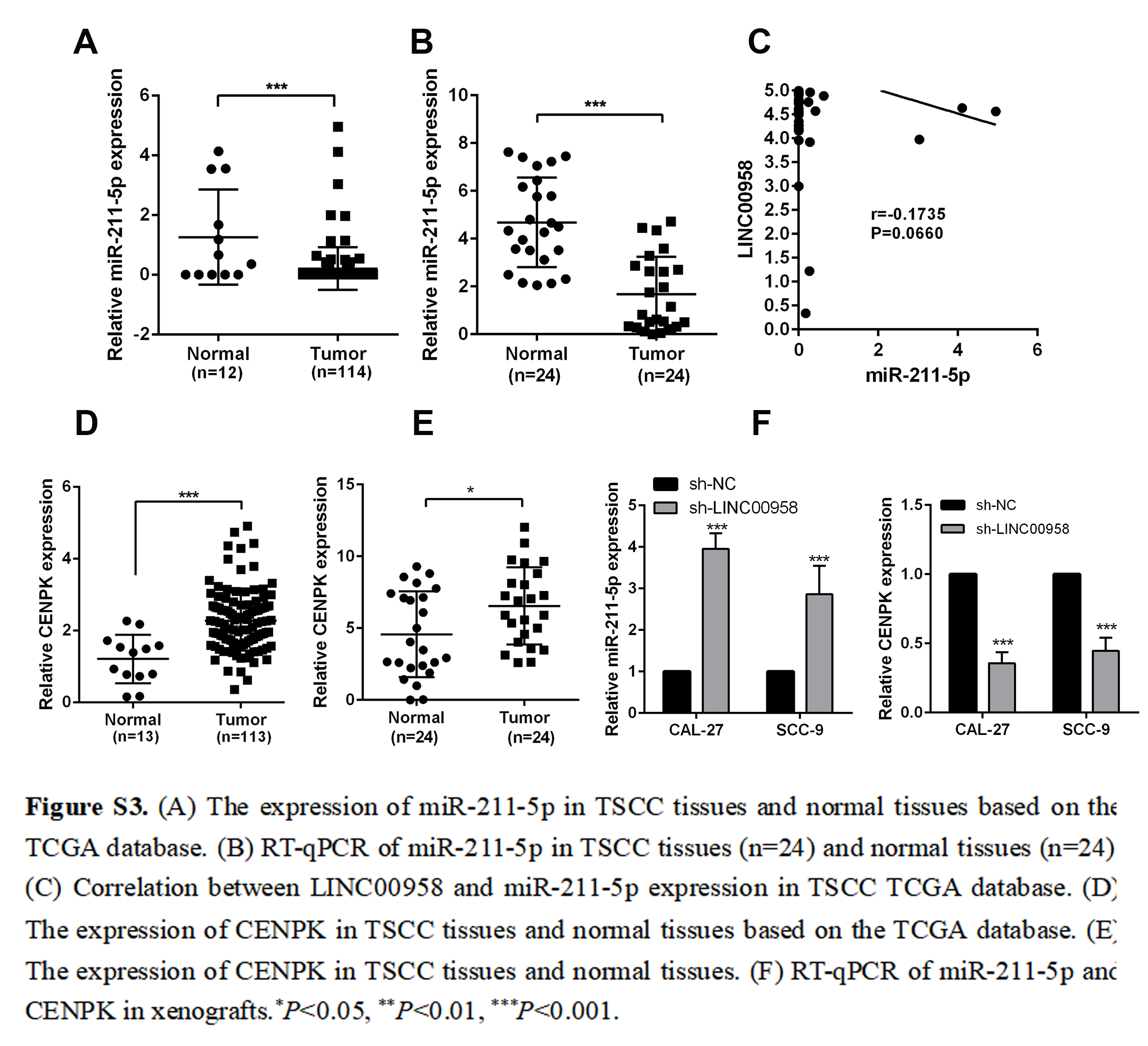

Supplement: Supplementary file 5 — Additional file 5. Figure S3. (A) The expression of miR-211-5p in TSCC tissues and normal tissues based on the TCGA database. (B) RT-qPCR of miR-211-5p in TSCC tissues (n=24) and normal tissues (n=24) (C) Correlation between LINC00958 and miR-211-5p expression in TSCC TCGA database. (D) The expression of CENPK in TSCC tissues and normal tissues based on TCGA database. (E) The expression of CENPK in TSCC tissues and normal tissues based on TCGA database. (F) RT-qPCR of miR-211-5p and CENPK in xenografts. *P<0.05, **P<0.01, ***P<0.001. [file 12935_2021_1808_MOESM5_ESM.jpg]

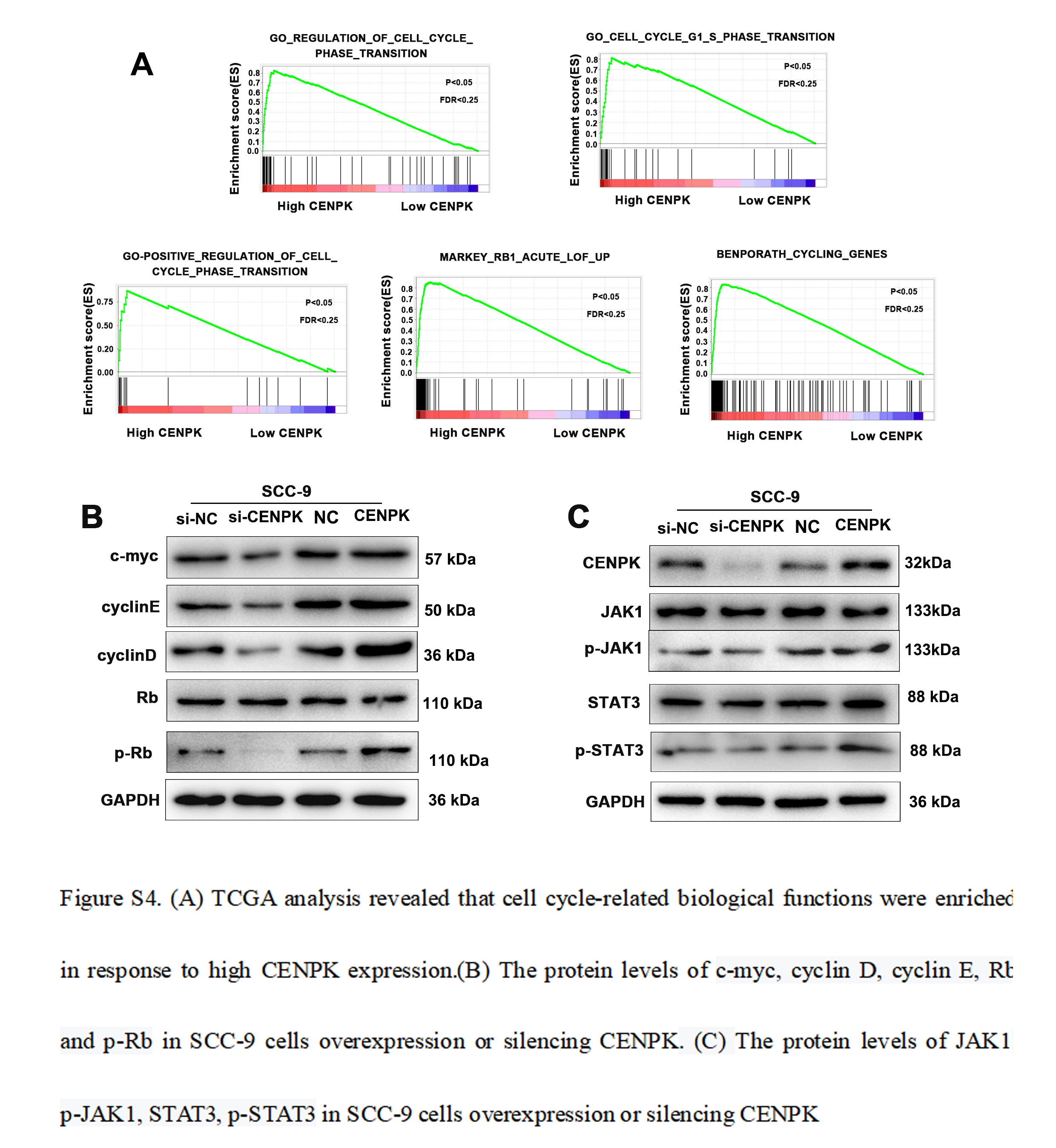

Supplement: Supplementary file 6 — Additional file 6. Figure S4. (A) TCGA analysis revealed that cell cycle-related biological functions were enriched in response to high CENPK expression. (B) The protein levels of c-myc, cyclin D, cyclin E, Rb and p-Rb in SCC-9 cells overexpression or silencing CENPK. (C) The protein levels of JAK1, p-JAK1, STAT3, p-STAT3 in SCC-9 cells overexpression or silencing CENPK. [file 12935_2021_1808_MOESM6_ESM.jpg]
