# Supplementary material for: LINC00958 promotes the proliferation of TSCC via miR-211-5p/CENPK axis and activating the JAK/STAT3 signaling pathway
Source: Cancer Cell Int. 2021 Mar 3;21:147. doi: 10.1186/s12935-021-01808-z (PMC7931557; doi:10.1186/s12935-021-01808-z)
Supplement: Supplementary file 3 — Additional file 3. Table S2. Sequences of PCR primers used in this study. [file 12935_2021_1808_MOESM3_ESM.docx]

**Table S2. Correlation of clinicopathological characteristics and gene( protein )expression in the patients**

| Variables | | No. of patients (n=113) | LINC00958 expression | | Chi-squared test p-value |
| --- | --- | --- | --- | --- | --- |
|  |  |  | Low | High |  |
| Age (yr) | |  |  |  | 0.958 |
|  | ＜45 | 27 | 14 | 13 |  |
|  | ≥45 | 96 | 42 | 54 |  |
| Gender | |  |  |  | 0.743 |
|  | male | 74 | 36 | 38 |  |
|  | female | 39 | 20 | 19 |  |
| Tumor invasion (T) | |  |  |  | . |
|  | T1 | 10 | 9 | 1 | 0.042* |
|  | T2 | 47 | 24 | 23 |  |
|  | T3 | 37 | 15 | 22 |  |
|  | T4 | 19 | 8 | 11 |  |
|  | T1+T2 | 57 | 33 | 24 | 0.110 |
|  | T3+T4 | 56 | 23 | 33 |  |
| Tumor grade(G) |  |  |  |  |  |
|  | G1 | 15 | 10 | 5 | 0.359 |
|  | G2 | 76 | 36 | 40 |  |
|  | G3 | 22 | 10 | 12 |  |
| Clinical stage | |  |  |  |  |
|  | Stage I | 12 | 9 | 3 | 0.110 |
|  | Stage II | 22 | 10 | 12 |  |
|  | Stage III | 25 | 15 | 10 |  |
|  | Stage IV | 54 | 22 | 32 |  |
|  | Stage I+II | 34 | 19 | 15 | 0.498 |
|  | Stage III+IV | 79 | 37 | 42 |  |
| Lymph nodes metastasis | |  |  |  | 0.219 |
|  | No | 59 | 33 | 26 |  |
|  | Yes | 54 | 23 | 31 |  |
| Distant metastasitc | |  |  |  | 0.993 |
|  | No | 112 | 55 | 57 |  |
|  | Yes | 1 | 1 | 0 |  |
| * p<0.05. Pearson's Chi-squared test(continuity correction) | | | | | |
