# Supplementary material for: LINC00958 promotes the proliferation of TSCC via miR-211-5p/CENPK axis and activating the JAK/STAT3 signaling pathway
Source: Cancer Cell Int. 2021 Mar 3;21:147. doi: 10.1186/s12935-021-01808-z (PMC7931557; doi:10.1186/s12935-021-01808-z)
Supplement: Supplementary file 7 — Additional file 7. Table S3. HALLMARK_IL6_ JAK_STAT3_SIGNALING Gene Set Enrichment. [file 12935_2021_1808_MOESM7_ESM.docx]

**Table3 HALLMARK_IL6_ JAK_STAT3_SIGNALING Gene Set Enrichment**

|  | PROBE | DESCRIPTION<br>(from dataset) | GENE SYMBOL | GENE_TITLE | RANK IN GENE LIST | RANK METRIC SCORE | RUNNING ES | CORE ENRICHMENT |
| --- | --- | --- | --- | --- | --- | --- | --- | --- |
| row_0 | IRF9 | na | null | null | 430 | 0.281712562 | -0.043442912 | Yes |
| row_1 | IL15RA | na | null | null | 503 | 0.264217824 | -0.010287358 | Yes |
| row_2 | IL12RB1 | na | null | null | 523 | 0.260289073 | 0.033958822 | Yes |
| row_3 | SOCS1 | na | null | null | 541 | 0.257000864 | 0.07803863 | Yes |
| row_4 | CBLB | na | null | null | 552 | 0.254064947 | 0.12313302 | Yes |
| row_6 | IL13RA2 | na | null | null | 831 | 0.211080536 | 0.14661156 | Yes |
| row_7 | OSMR | na | null | null | 888 | 0.203541875 | 0.17203397 | Yes |
| row_8 | IL11RA | na | null | null | 890 | 0.20308052 | 0.209639 | Yes |
| row_11 | IFNA1 | na | null | null | 991 | 0.18937254 | 0.29611543 | Yes |
| row_12 | LIFR | na | null | null | 1063 | 0.182744011 | 0.3143178 | Yes |
| row_13 | IL2RA | na | null | null | 1200 | 0.168781653 | 0.31542018 | Yes |
| row_14 | TPO | na | null | null | 1224 | 0.164455146 | 0.34092298 | Yes |
| row_15 | CSF2 | na | null | null | 1267 | 0.160677135 | 0.3614838 | Yes |
| row_16 | CSF3 | na | null | null | 1339 | 0.153387204 | 0.37421784 | Yes |
| row_17 | IL22RA2 | na | null | null | 1373 | 0.150178149 | 0.39483058 | Yes |
| row_18 | IFNE | na | null | null | 1375 | 0.150018111 | 0.42255163 | Yes |
| row_19 | IFNG | na | null | null | 1517 | 0.138627738 | 0.41692185 | Yes |
| row_20 | PIK3CD | na | null | null | 1695 | 0.124670558 | 0.40066192 | Yes |
| row_21 | IL11 | na | null | null | 1719 | 0.122594617 | 0.4183673 | Yes |
| row_22 | IL24 | na | null | null | 1883 | 0.10789267 | 0.40210503 | Yes |
| row_23 | IL19 | na | null | null | 1929 | 0.103863947 | 0.411414 | Yes |
| row_24 | IL7R | na | null | null | 2011 | 0.095903426 | 0.41120982 | Yes |
| row_25 | IL21R | na | null | null | 2018 | 0.095027991 | 0.42757243 | Yes |
| row_26 | EPO | na | null | null | 2110 | 0.086404346 | 0.4233682 | No |
| row_27 | SPRY4 | na | null | null | 2208 | 0.077055298 | 0.41608408 | No |
| row_28 | IL12RB2 | na | null | null | 2359 | 0.061351486 | 0.39405236 | No |
| row_29 | IFNK | na | null | null | 2533 | 0.04540237 | 0.36391932 | No |
| row_30 | CSF3R | na | null | null | 2968 | -6.34E-04 | 0.2672272 | No |
| row_31 | IL26 | na | null | null | 3050 | -0.009408426 | 0.25091147 | No |
| row_32 | PRLR | na | null | null | 3052 | -0.009636899 | 0.2524835 | No |
| row_33 | CNTFR | na | null | null | 3257 | -0.031288482 | 0.2128064 | No |
| row_34 | IL10 | na | null | null | 3684 | -0.073525906 | 0.13147652 | No |
| row_35 | IL5RA | na | null | null | 3787 | -0.084466621 | 0.12445762 | No |
| row_36 | IL22 | na | null | null | 3819 | -0.088268518 | 0.1339845 | No |
| row_37 | OSM | na | null | null | 4002 | -0.118971244 | 0.115547635 | No |
